# Industrially Produced Fe- and Mn-Based Perovskites: Effect of Synthesis on Reactivity in Three-Way Catalysis: Part 1

**DOI:** 10.1021/acsomega.1c02133

**Published:** 2021-09-14

**Authors:** Elena Brusamarello, Cataldo Blonda, Cristina Salazar-Castro, Andrea Eva Pascui, Paolo Canu, Antonella Glisenti

**Affiliations:** † Department of Chemical Sciences, University of Padova, Via F. Marzolo, 1, 35131 Padova, Italy; ‡ L’Urederra Foundation, Perguita Industrial Area, No. 1 Street, CP, Los Arcos, 31210 Navarra, Spain; § Johnson Matthey Technology Centre, Blount’s Court Sonning Common, RG4 9NH Reading, U.K.; ∥ Department of Industrial Engineering, University of Padova, Via F. Marzolo, 9, 35131 Padova, Italy; ⊥ CNR-ICMATE, INSTM, Via F. Marzolo, 1, 35131 Padova, Italy

## Abstract

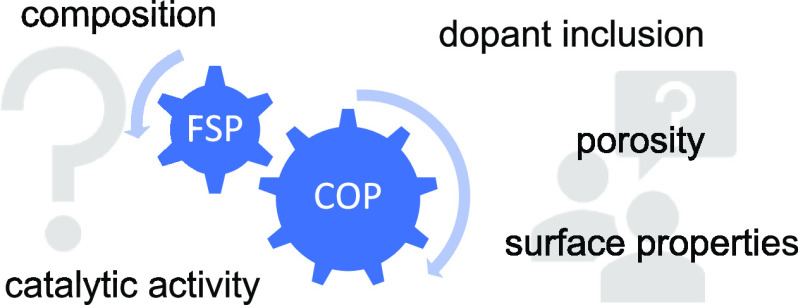

La_0.6_Ca_0.2_Fe_0.8_Cu_0.2_O_3_, undoped (LF) and Ca, Cu-doped (LCFC), powders, obtained by different industrial procedures, are compared to evaluate reproducibility and scale-up in different industrial synthetic approaches: flame spray pyrolysis (FSP) and coprecipitation (COP). Also the effects of varying composition (doping) and FSP process variability are considered as comparative studies on morphological, crystallographic, redox and compositional properties, and functional activity. A model reaction (CO + NO) and reactions with an automotive exhaust mixture were carried out. Unexpected results on the effectiveness of doping for catalytic activity emerged. Samples with the same compositions proved to be significantly affected by the synthesis, with variability within the same process. The activity of LCFC COP is comparable to the FSP analogue, at stoichiometric conditions, notwithstanding differences highlighted by characterization. In an oxygen-deficient mixture, LCFC-COP yields higher NO reduction and CO oxidation activity than LCFC-FSP. The absence of Ca in the lattice was unexpectedly beneficial. The doping effectiveness must be carefully checked for large-scale production.

## 1 Introduction

The development of innovative catalysts for pollutant control focused on perovskites as versatile materials, which allows the incorporation of different cations in their structure to tune activity and selectivity.^1^ Adding inexpensive and largely available, catalytically active transition-metal cations can represent a novel approach for three-way catalysis (TWC)^2^ that allows minimizing noble metal utilization. TWC is the commonly used exhaust emission control technology to deal with toxic pollutants in exhaust gases from automotive engines. In particular, they are optimized to combine oxygen with carbon monoxide and unburned hydrocarbon to produce carbon dioxide and water and, simultaneously, to reduce oxides of nitrogen (NO_*x*_) to gaseous nitrogen.

Perovskite oxides have a general formula ABO_3_, in which the A-site ion is usually an alkaline earth or rare-earth element, whereas the B-site ion is usually a transition-metal ion. The aliovalent doping of the perovskite A and B sites induces the formation of structural defects and different oxidation states, together with cationic redox couples.^3−6^

A relevant problem, when developing complex formulations, is the synthesis of the catalysts both in terms of reproducibility and scale-up. There are several routes for the synthesis of perovskites.^7−13^ Coprecipitation (COP) and flame spray pyrolysis (FSP) are among the more industrially adopted methods. In COP, the precipitation of the hydroxide precursors is induced by pH change and is followed by calcination treatments to obtain the final phase. FSP is appreciated for scale-up, being a continuous process in which the precursor solution is kept at high temperatures for a very short time to assure the formation of the desired phase while avoiding the decrease of the specific surface area. The first reports of perovskite synthesis using FSP were by Brewster and Kodas in 1997 who prepared BaTiO_3_ by spraying an aqueous Ba acetate/Ti lactate feed into a H_2_/air flame.^14^ This was followed by Leanza et al. who prepared La_1–*x*_M_*x*_CoO_3_ (M = Ce, Eu) by spraying an aqueous metal acetate/nitrate/citrate feed into a H_2_/O_2_ flame.^15^

This is the first part of the article, which comprises a second one and whose aim is to investigate the effects of industrial scale-up on functional properties of the catalysts, and therefore two different materials are considered: Fe-based Cu-doped and Mn-based K-doped perovskites to shine light on the differences and similarities between different compositions when subjected to different synthetic approaches. We indeed suppose that the differences in the synthesis carried out may be more influent on the results when the composition is more complex, depending also on the cations involved.

In this first part of the paper, we compare undoped (LF) and Ca, Cu-doped (LCFC) under stoichiometric lanthanum ferrites synthesized by COP and FSP. The interest on ferrite perovskites has recently grown in literature.^16−29^ It was found that oxidation reactions and thermal stability of the materials are enhanced in the case of La-deficient compositions.^30−33^ Moreover, computational calculations also concluded that calcium inclusion can enhance oxygen conductivity in LaFeO_3_.^34^

In the second part of the present paper, an analogous work is presented regarding Mn-based K-doped perovskites, prepared with the same approach as follows.

Catalysts obtained by FSP and by COP have been compared considering TWC as a potential application (automotive exhaust treatment, in fact, is a very demanding form of catalysis). Doped ferrites and manganites have been considered because of their good fresh activity in TWC reactions, low cost, and absence of noble metals.^30,32,35^ Given that FSP is a continuous process, the samples obtained in successive stages of the synthesis procedure have been characterized.

The catalytic activity of the samples (both in the first and second parts of the paper) in TWC reactions is correlated with X-ray diffraction (XRD), X-ray photoelectron spectroscopy (XPS), temperature-programmed reduction (H_2_-TPR), Brunauer–Emmett–Teller (BET) surface area measurements, scanning electron microscopy (SEM), and energy-dispersive X-ray analysis (EDX) results.

## 2 Experimental Section

### 2.1 Synthesis

#### 2.1.1 Flame Spray Pyrolysis

The FSP perovskites were produced by FSP technology, with a small-scale flame reactor owned by Lurederra which allows a production capacity of 0.1 kg/h. The FSP technology consists of a simple one-step aerosol combustion process where a mixture of metal precursors, dissolved in an appropriate solvent (specifically xylene) is sprayed with an oxidizing gas (specifically air) into a high-temperature flame zone where the aerosol droplets generated are individually evaporated and oxidized, turning the mixture of metal precursors into nanosized particles with a perovskite structure. Properties such as high purity, low aggregation, and small nanoparticle size are typically obtained following this process and can be controlled depending on the operational parameters, namely, precursor feed flow, amount of the dispersant gas, nozzle pressure, flame morphology, and so forth.^14,15^

The production of LCFC has been distributed over several days, to assess the variability of the product and process after several operation phases, including cycles of start-up and shut-down, and variable duration of the stable operations. The detailed description of the experimental conditions for the sample collection (categorized as LCFC A, B, C, D, and E) is reported in the Supporting Information document.

#### 2.1.2 Coprecipitation

For the synthesis of LCFC COP, the following procedure was followed: KOH (>85%, 750 g, 11.4 mol) was dissolved in water (7 L) and the solution was stirred and heated to 60 °C. Lanthanum nitrate hexahydrate (649.5 g, 1.5 mol), calcium nitrate (118 g, 0.5 mol), iron nitrate nonahydrate (808 g, 2 mol), and copper nitrate trihydrate (120.8 g, 0.5 mol) were dissolved in water to give 1.5 L of the total volume of solution. The salt solution was added to the base at 10 mL/min. When the addition was completed, the precipitate was aged with stirring for 30 min at 60 °C. The material was collected by vacuum filtration, washed to remove adsorbed ions, and dried at 105 °C. The sample was fired at 700 °C for 2 h in air to form the perovskite phase.

### 2.2 Characterization

XPS measurements were carried out with a PerkinElmer 5600 ci Multi Technique System. A spectrometer is calibrated by assuming the binding energy (BE) of the Au 4f_7/2_ line to be 84.0 eV with respect to the Fermi level. Both extended spectra (survey, 187.85 eV pass energy, 0.5 eV/step, 0.05 s/step) and detailed spectra (for La 3d, O 1s, C 1s, Fe 2p, Ca 2p, Cu 2p—23.5 eV pass energy, 0.1 eV/step, 0.1 s/step) are collected with a standard Al Kα source working at 250 W. The standard deviation in BE values of the XPS line is 0.10 eV. The atomic percentage, after a Shirley-type background subtraction,^36^ is evaluated by using the PHI sensitivity factors.^37^ The peak positions are corrected for the charging effects by considering the C 1s peak at 284.8 eV and evaluating the BE differences.^38^ All the fitting procedures are carried out on normalized spectra.

XRD analyses are performed with a Bruker D8 ADVANCE diffractometer with a Bragg–Brentano geometry using a Cu K_α_ radiation (40 kV, 40 mA, *k* = 0.154 nm).

Field-emission SEM and energy-dispersive X-ray spectroscopy measures are carried out on a Zeiss Supra 40VP. Both morphological and compositional analyses are carried out setting the acceleration voltages at 20 kV.

TPR is performed with an AutoChem II 2920 Micromeritics, equipped with a TCD detector. TPR measurements are carried out in a quartz reactor by using 50 mg of the sample and heating from RT to 900 °C at 10 °C/min under a constant flow of H_2_ 5% in Ar. TPR samples were previously outgassed with He (50 mL·min^–1^) at RT. The surface area of all the samples is determined by an Asap 2020 Plus from Micromeritics. The measurements are carried out at the liquid nitrogen temperature (77 K). The specific surface area is calculated using the BET equation. Prior to N_2_-sorption, all the samples are degassed at 200 °C for 16 h.

### 2.3 Activity Tests

Two series of catalytic activity tests were carried out, at atmospheric pressure, on simple and more complex feed mixtures. All the inlet compositions and GHSV data are summarized in Table 1.

**Table 1:** Feed Composition of all the Measurements (λ = O_2_ Fed/O_2_ Stoich = [O_2_]/(0.5 [CO] + 0.5 [H_2_] + 2 [CH_4_] + 4.5 [C_3_H_6_] +5 [C_3_H_8_] – 0.5 [NO]))

inert	CO_2_ (%)	H_2_O (%)	O_2_ (%)	CO (%)	NO (%)	H_2_ (%)	CH_4_ (ppm)	C_3_H_6_ (ppm)	C_3_H_8_ (ppm)	Λ		*m*_cat_ (mg)	flow rate (smL/min)	WHSV (smL/h)/g_cat_
He				1	1							50	100	150000
He	15	10	0.777	0.7	0.1	0.233	230	450	230	1.0	stoich	200	200	60000
He	15	10	0.609	0.9	0.1	0.300	300	600	300	0.6	rich	200	200	60000

The first set of measurements is based on a model reaction, NO + CO. Stoichiometric CO and NO (1% each) are fed with Ar to a quartz reactor (6 mm ID) hosting a packed bed of the catalyst as the powder (50 mg); the temperature was monitored by a thermocouple inserted right upstream of the bed. The inert carrier was always He (Table 1). The flows were controlled by thermal mass flowmeters (Vögtlin Instruments). The temperature of the bed was varied between RT and 500 °C with a stepped temperature program. The reaction products were monitored with an Agilent 7890A gas chromatograph, equipped with a TCD detector. The columns are a molecular sieve 13X (60/80 mesh, 1.8 m) and a Porapak Q (1.8 m). The calibration was done using standard gases containing known concentrations of the components.

The second set of catalytic activity measurements aimed at resembling the actual conditions of an automotive exhaust. The gas feed composition is reported in Table 1. In addition to a larger number of species, 10 vol % of steam and 15 vol % of CO_2_ were always used, reflecting actual conditions, quite challenging for the catalysts. A different quartz tube flow reactor, 8 mm ID, was used, in which the catalytic bed is placed. The experimental setup has been already described.^39^ Shortly, the electrically heated quartz reactor contains a shallow bed of the catalyst as powder (200 mg of sample), sieved in the size 250–350 μm. The temperature is monitored upstream the bed; it was varied between RT and 600 °C. The flow rates were controlled by mass flow meters (Brooks Instruments and Bronkhorst High-Tech). Steam was generated by an isothermal bubbler. Each catalyst was tested at both fuel-rich and stoichiometric conditions; stoichiometric O_2_ is determined from the total oxidation of the fuels (CO, H_2_, and HCs), accounting also for the O_2_ expected from NO reduction. The vapor in the outlet gas was condensed and the dry mixture analyzed. The following gases were determined: H_2_, CO_2_, O_2_, N_2_, CO, and HCs by GC (Agilent 7820, with Porapak Q and MS5A using TCD and FID in series), at a frequency of 1/9 sample/min. CO, CO_2_, NO, and NO_2_ are measured by FTIR (Shimadzu IRTracer-100), at a frequency of 1 sample/min. CO and CO_2_ reported in the results are taken from GC, which from past experience has shown to be more accurate than IR in detecting these gases.

The standard testing sequence in the second set of measurements is as follows: (1) heating the catalyst at 10 °C/min to 600 °C in air, (2) 2 h of pre-conditioning at 600 °C in air, (3) 2 h of conditioning at 600 °C with the reaction mixture, (4) slow temperature decrease (−2 °C/min) to measure the catalyst activity at different temperatures, down to 90 °C, a temperature at which no catalytic activity is detected. It has been verified that a selected cooling rate of −2 °/min is sufficiently slow to achieve the steady-state operation of the catalyst, at each temperature scanned. Because the test is basically conducted at a small isothermal plateaux, the exothermicity of the reactions involved (such as CO, H_2_, and HC oxidations) does not represent a noticeable issue for the accuracy of the testing.

The simple mixture was tested at rising temperatures, on a catalyst without any pretreatment (to understand the effect of the surface composition and active sites), while the complex mixture was tested from 600 °C during controlled cooling, after the pretreatment of the catalyst in air, at high temperatures (following protocols suggested by industries for automotive catalysts). Reactant conversion and product yield are defined with molar fractions *x* (mixtures are very diluted) as
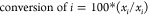

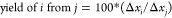


## 3 Results and Discussion

The sample compositions and features have been summarized in Table 2.

**Table 2:** Composition and Specific Surface Area of the Samples Analyzed

sample—synthesis	composition	specific surface area (m^2^/g)
LCFC A—FSP	La_0.6_Ca_0.2_Fe_0.8_Cu_0.2_O_3_	61.1
LCFC B—FSP	La_0.6_Ca_0.2_Fe_0.8_Cu_0.2_O_3_	59.1
LCFC C—FSP	La_0.6_Ca_0.2_Fe_0.8_Cu_0.2_O_3_	57.0
LCFC D—FSP	La_0.6_Ca_0.2_Fe_0.8_Cu_0.2_O_3_	59.0
LCFC E—FSP	La_0.6_Ca_0.2_Fe_0.8_Cu_0.2_O_3_	60.8
LFC—FSP	La_0.7_Fe_0.8_Cu_0.2_O_3_	32.5
LCFC—COP	La_0.6_Ca_0.2_Fe_0.8_Cu_0.2_O_3_	22.0

### 3.1 Characterization

Significant differences have been found comparing La_0.6_Ca_0.2_Fe_0.8_Cu_0.2_O_3_ obtained by FSP (LCFC FSP variant E, among the 5 produced and compared in the Supporting Information) and by coprecipitation (LCFC COP). The results of the characterization of the samples obtained during different phases of the FSP production is reported in the Supporting Information.

To help understand the effect of Ca-doping, La_0.6_Ca_0.2_Fe_0.8_Cu_0.2_O_3_ was compared with the Ca-free La_0.7_Fe_0.8_Cu_0.2_O_3_ (LFC FSP) both obtained by FSP.

**Figure 1:**
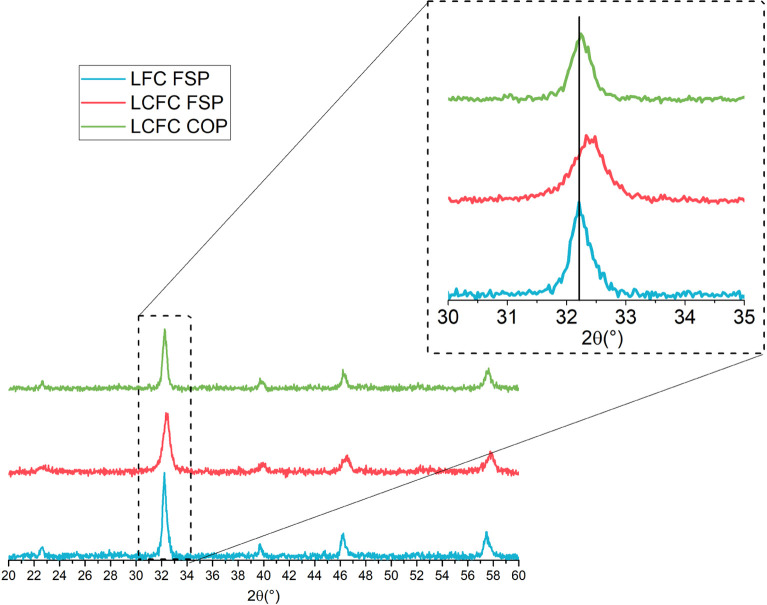
XRD pattern of the samples LCF FSP, LCFC FSP, and LCFC COP.

The XRD patterns, as shown in Figure 1, show the expected perovskite phase (orthorhombic lattice symmetry). The intense XRD peaks at 2θ values of 32.21, 39.65, 46.25, and 57.44° correspond to the typical (121), (220), (202), and (240) reflection plans, thus relating it to the orthorhombic crystal structure with a space group Pbmm (JCPDS PDF 37-1493). The non-doped sample LFC obtained by FSP and the doped sample obtained by COP, LCFC COP, are very similar in terms of crystalline composition. The LCFC FSP pattern, in contrast, is shifted toward higher 2θ values (Figure 1, inset) due to the contraction of the unit cell volume consequent to the inclusion of Ca. The ionic radii of La(III) and Ca(II) are very near and no significant variation of the unit cell is expected as a consequence of the substitution; the Fe(IV) small radius (*r*_Ca(II)_ = 134 pm, *r*_La(III)_ = 136 pm) can account for the cell contraction (*r*_Fe(III)_ = 64.5 pm, *r*_Fe(IV)_ = 58.5 pm). The formation of Fe(IV) was observed to be induced by Ca doping^30,35,40^ and confirmed in perovskites by Mossbauer spectroscopy.^41^ Beside Fe(IV), oxygen vacancies can also result from electric charge compensation^42^ and cause the lattice restriction. Barbero et al. observed that the formation of oxygen vacancies becomes significant for *x* > 0.2 in stoichiometric La_1–*x*_Ca_*x*_FeO_3_^43^ but the A-site substoichiometry and the presence of Cu is also expected to favor their formation.^30,32^ The shift of the XRD signals suggests the un-complete inclusion of Ca in the perovskite lattice for the sample obtained by COP and its inclusion in the FSP one. Rietveld refinement studies on similar samples corroborate this hypothesis.^30^

In Figure S1 (Supporting Information), XP spectra are reported. O 1s spectra indicate two distinct contributions, lattice oxygen (at about 529 eV) and surface oxygen species (532.0 eV).^4,38,41,44−53^ A fitting procedure reveals that FSP samples are characterized by a higher amount of surface oxygen species with respect to the COP ones, Figure S2 in Supporting Information.

La 3d_5/2_ and La 3d_3/2_ are consistent with the presence of La(III) (shake-up signals at 837.3 and 838.5 eV), and La(OH)_3_ and LaOOH (about 835 eV) are observed (833.5 for La 3d_5/2_ in perovskites).^39,41,44−56^

The Ca 2p region is identical for the two Ca-doped samples. Fe 2p peaks (710.5 eV) are a characteristic of Fe(III).^14,21,30,32^ The Cu 2p_3/2_ XP spectrum is centered at 933.5 eV, a position characteristic of Cu in oxides; the presence of Cu(II) is confirmed by the shake-up at 941.8 eV.^4,53^

Oxygen is over stoichiometric and is more abundant in the FSP samples. Table S2 shows that in the LFC sample Fe and La are surface segregated, whereas Cu is less than expected (even if more abundant in XPS than in EDX). The same observation applies to samples LCFC FSP and COP with regard to La, but Ca doping causes a further decrease of surface Cu. Only in the COP sample (and with EDX), the amount of Cu gets closer to the expected value. Ca is present in significantly lower amounts in the COP catalyst.

**Figure 2:**
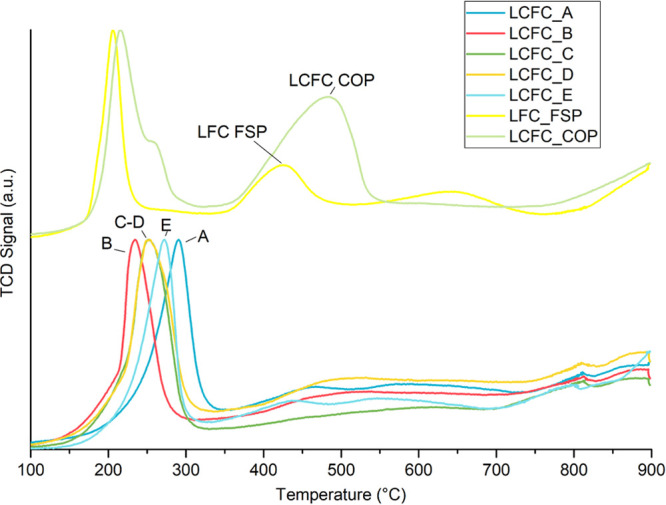
H_2_-TPR profile for LFC FSP, LCFC COP, and LCFC FSP obtained during different production moments (A–E).

TPR curves for each sample have been compared in Figure 2. Three peaks are identified at 205–278 °C [Cu(II) to Cu(0)] and 422–481 °C, Fe(IV) to Fe(III), and about 650 °C, [Fe(III) to Fe(II)].^9,35^ The two contributions of iron oxide reduction are very weak in the LCFC FSP sample with respect to LFC FSP (2–4% of the value expected from the composition to be compared with the 65% of the Ca-free catalyst). This is evidence for the stabilization of Fe(III) and Cu(II) (whose reduction temperature increases by about 100 °C) in the lattice structure or of a low ion mobility.

Calcium doping has proved to be effective in decreasing the reducibility of the FSP sample. Wu et al.^30^ compared the behavior of stoichiometric and under stoichiometric, doped and un-doped LaFeO_3_. No significant reduction is observed in the stoichiometric sample, whereas a broad signal due to the reduction of segregated α-Fe_2_O_3_ is observed in the under stoichiometric one. After doping the reduction of iron oxide is not observed anymore and the reduction of Cu(II) to Cu(0) is evident. The insertion of calcium into the perovskite stabilizes Cu inside the unit cell unfavoring its segregation as CuO and its reduction. In COP sample, the apparently incomplete inclusion of Ca in the structure seems to confirm this hypothesis: a double spiked peak that accounts for the reduction of two distinct species of Cu(II) agrees with the presence of segregated easily reducible CuO and less reducible Cu(II) inserted into the perovskite unit cell. In fact, the low-temperature contribution seems to agree with the FSP sample without Ca, whereas the high-temperature contribution is consistent with the LCFC FSP catalyst (E). The ratio between these two contributions is around 1:3 (Table 3).

**Table 3:** H_2_-TPR Results for Samples the FSP and COP Catalysts^a^

	*T*_max_ (°C)	mol H_2_ consumed/expected	assignment	Fe(IV)/((Fe(IV) + Fe(III))
LCFC FSP				0.47
low *T* peak	272	0.65	Cu(II)–Cu(0)	
broad signal	450–650	0.02	Fe(IV)–Fe(III); Fe(III)–Fe(II)	
LFC FSP				0.27
low *T* peak	206	0.54	Cu(II)–Cu(0)	
broad signal	450–650	0.65	Fe(IV)–Fe(III); Fe(III)–Fe(II)	
LCFC COP				0.42
low *T* peak	216–250	1.02	Cu(II)–Cu(0)	
broad signal	450–650	0.18	Fe(IV)–Fe(III); Fe(III)–Fe(II)	

a The third column (mol H_2_ consumed/expected) refers to the experimental and theoretical amount of H_2_ to be consumed during the TPR, in correlation with the stoichiometric composition of the samples.

SEM images are reported in S3. For the FSP materials, the pictures highlight the presence of the combustion residues (small white particles) and a homogeneous morphology with the dispersed particles. The LCFC COP sample reveals a more compact morphology with larger aggregates.

The catalyst produced by coprecipitation, LCFC COP (Table 1), shows a specific surface area of 22 m^2^/g, about one-third of the corresponding catalyst obtained by FSP, consistently with a more compact morphology; this can be due to the harsh calcination treatment. The different preparation procedures play a role in: a Ca, Cu-doped perovskite of identical composition but obtained by a citric acid procedure a specific surface area of 21.3 m^2^/g was obtained. The specific surface area is mainly affected by doping: in the LCFC FSP, indeed, it is around 60 (57.0–61.1 m^2^/g in the samples obtained at different synthesis steps, see in the following) whereas it is 32.5 m^2^/g in LFC FSP. This was attributed, by Andoulsi et al.^57^ to the increased nucleation sites resulting from higher stacking fault energy due to calcium incorporation into the perovskite lattice.^58^

#### 3.1.2 Catalytic Activity

**Figure 3:**
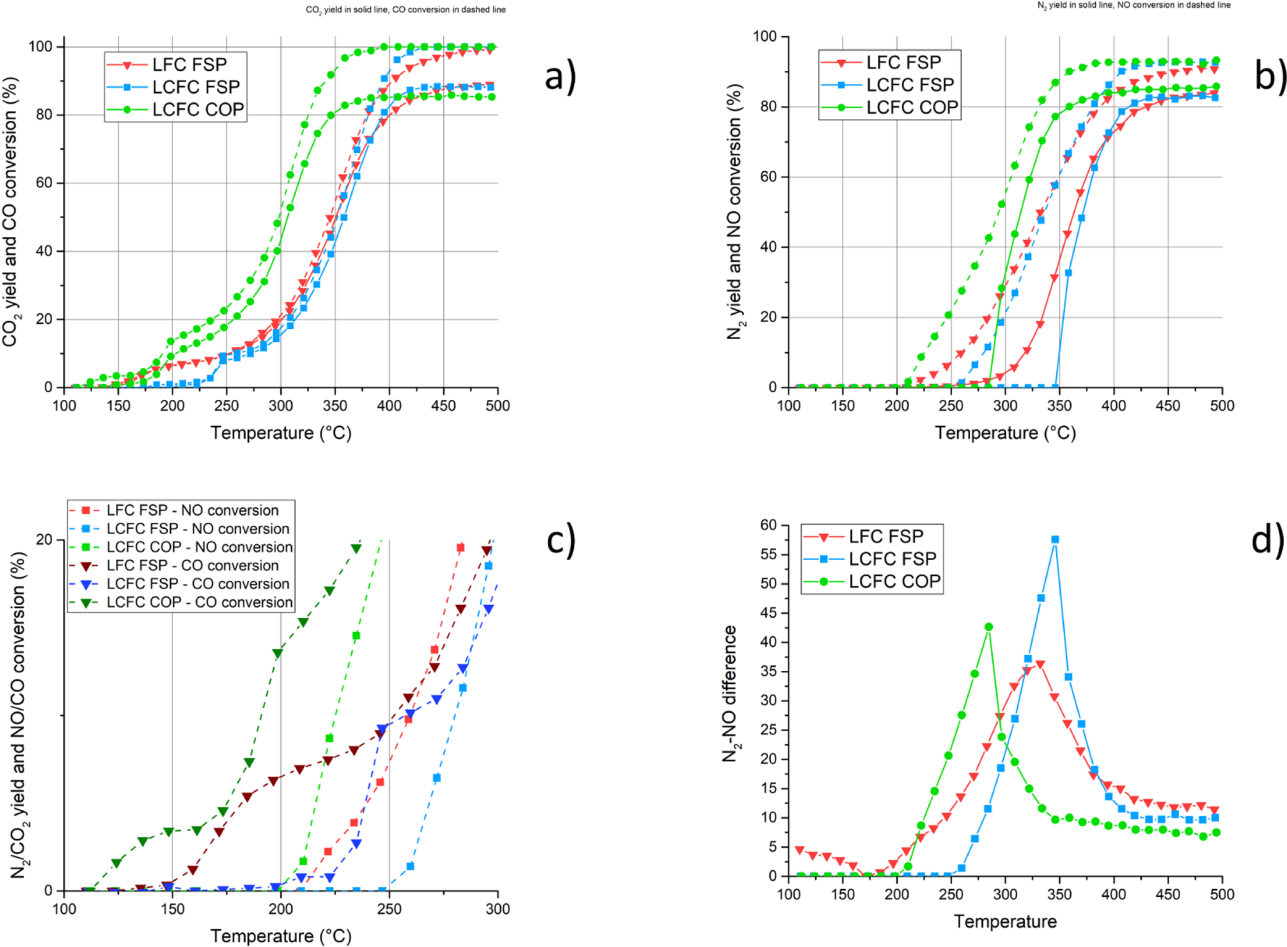
CO–NO mixture reactivity for samples LFC FSP, LCFC FSP, and LCFC COP. (a) CO conversion (dashed) and CO_2_ yield (solid); (b) NO conversion (dashed) and N_2_ yield (solid); and (c) N_2_/CO_2_ yield and NO/CO conversion between 100 and 300 °C. (d) N_2_O determined by the difference by NO conversion and N_2_ yield.

**Figure 4:**
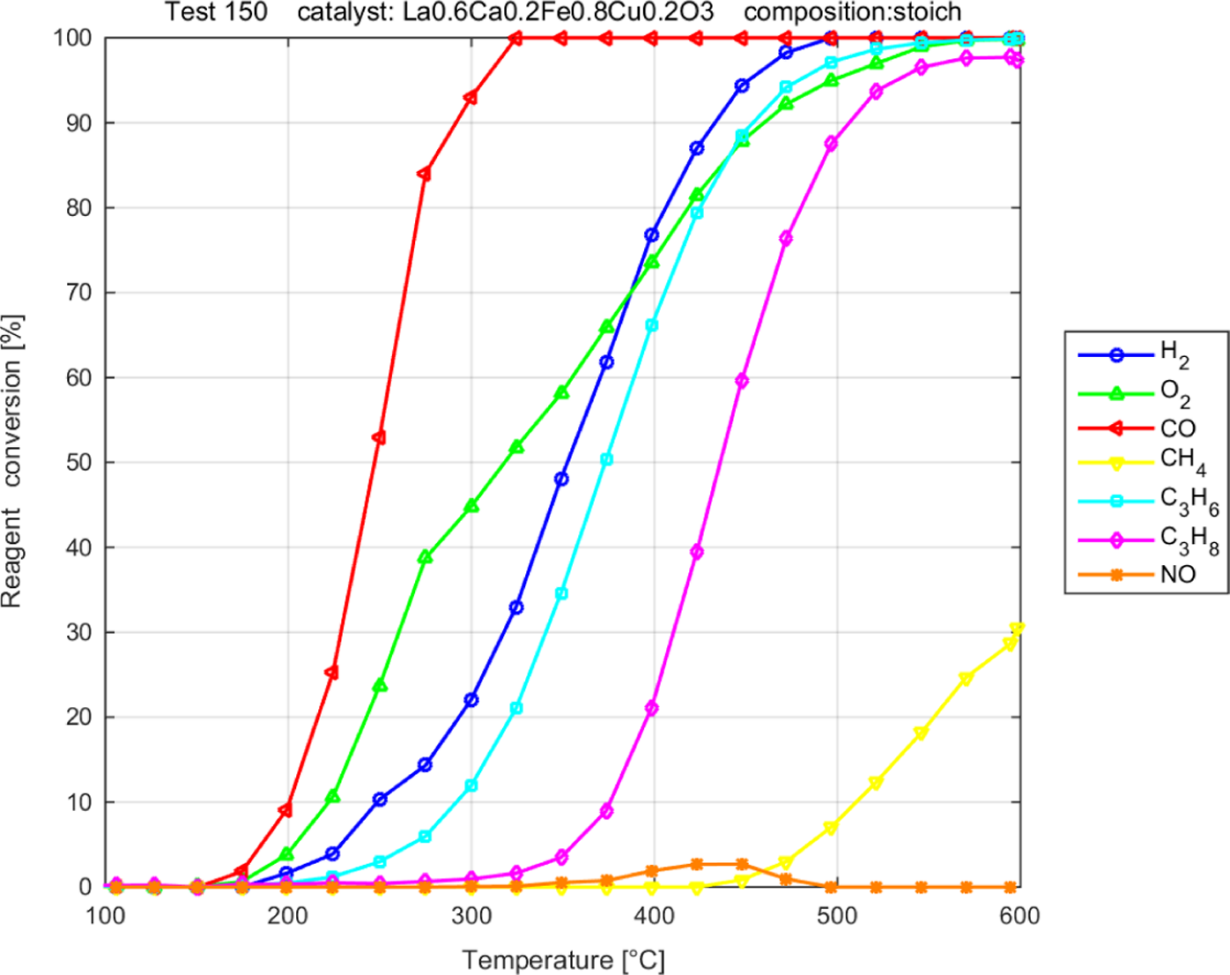
Activity of LCFC COP. Stoichiometric TWC mixture.

**Figure 5:**
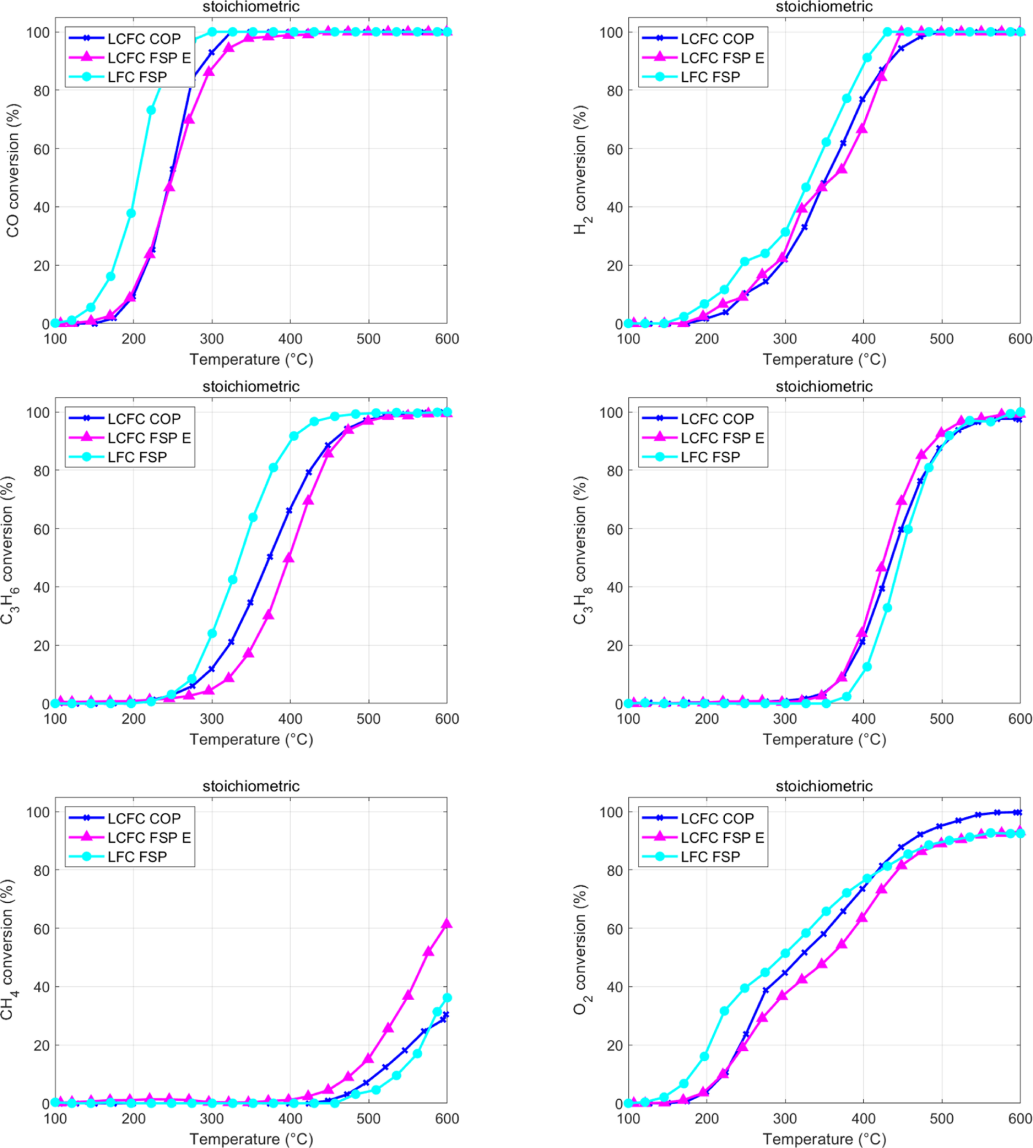
LCFC FSP and LCFC COP. Stoichiometric TWC mixture.

**Figure 6:**
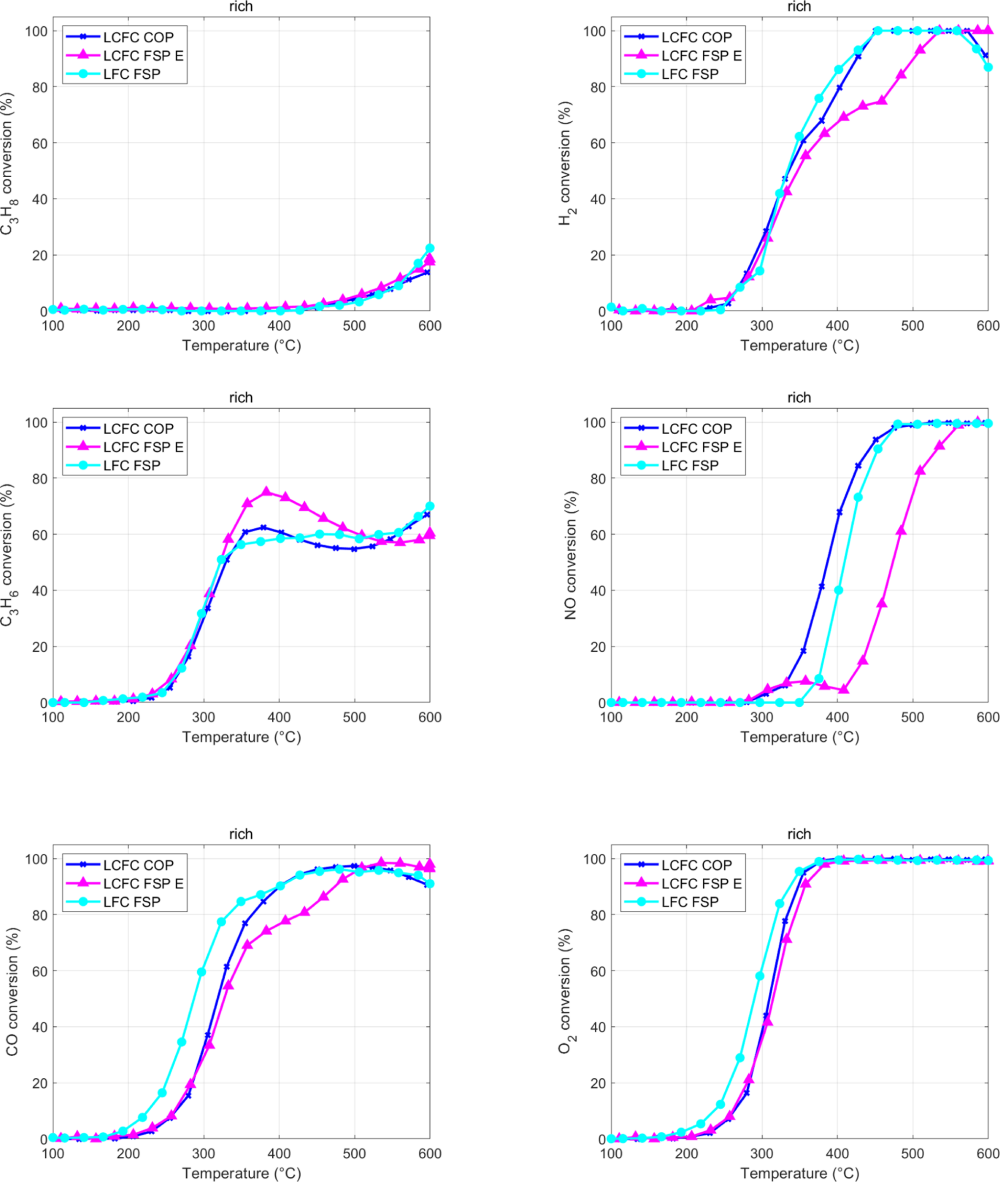
Activity of LFC FSP, LCFC FSP, and LCFC COP. Rich TWC mixture.

Each sample has been tested for CO-assisted NO reduction (Figure 3) and with a complex mixture simulating the automotive exhaust at rich and lean conditions (Figures 4–6).

The light-off temperature for CO oxidation and NO reduction is quite low for a non-PGM catalyst, approx. 250 °C, and is lower for LCFC COP; this is the best sample being able to approach the full conversion of CO and NO below 400 °C. Both samples from FSP (LFC FSP and LCFC FSP) behave very similarly, despite the different dopings and the differences highlighted by the characterization. These preliminary results suggest that the catalyst characterized by higher activity, in spite of the lower specific surface area, is the one obtained by coprecipitation.

CO conversion starts at 100 °C lower temperatures with respect to NO. It is widely accepted that CO oxidation can proceed via a Mars–van Krevelen mechanism^59^ using O from the material. CO molecularly adsorbs on Lewis surface acidic sites and reacts with oxygen species near the neighbors of the Lewis acidic sites, releasing carbon dioxide that desorbs. The result is a partially reduced site (oxygen vacancy) that may be re-oxidized by gas phase oxygen (if available) by bulk oxygen (if mobility is enough) or be the site for another CO chemisorption. In the catalyst without or with a small amount of Ca, this phenomenon is more evident.

The activity profiles suggest that CO conversion evolves with a change in the mechanism around 200–250 °C. At this temperature, the conversion of NO also begins, suggesting that NO contributes to the CO oxidation.

The comparison between the CO conversion and CO_2_ yield curves suggests that, particularly at temperatures higher than 350–400 °C, part of the carbon dioxide remains on the catalyst surface as an adsorbate. The insertion of calcium into the cell modifies the catalytic activity at lower temperatures: in LFC FSP the light-off temperature is around 150 °C, in LCFC FSP it is around 225 °C; moreover, the start of the light-off curve is steeper. For LCFC COP, the light-off temperature is around 125 °C. For LCFC FSP, a step-like increment is observed (around 200 °C) when the oxidation starts involving NO. The N_2_ production starts at higher temperatures than the NO consumption and it remains always less than that expected from NO reduction. This suggests that some N_2_O (not measured but calculated) might form at lower temperatures; although N_2_O is evidently less at higher temperatures, there is always some in the products.

The higher reactivity of the COP sample can be related to the higher amount of iron observed on the surface and the lower amount of oxygen, which could favor the formation of oxygen vacancies capable of activating NO and chemisorbing CO. The relevance of Fe(IV) on the catalytic activity of LaFeO_3_-based perovskites was already underlined.^30,35,40,43^

The activity of these catalysts under a complex TWC mixture, either stoichiometric or rich, is reported in Figures 4–6.

Figure 4 shows the conversion of all the reactants for LCFC COP at stoichiometric conditions, to highlight the sequential onset of reactivity. The sequence does not change for the other catalysts. CO is always oxidized first and then H_2_, followed by HCs with CH_4_ seldom activated.

Materials that differ by synthesis and Ca doping are compared in terms of single-reactant conversion with the stoichiometric mixture, as shown in Figure 5. All the materials are good oxidation catalysts, sequentially activating the oxidation of CO (between 120 and 170 °C on the different materials), H_2_ (∼170–200 °C), unsaturated (∼250 °C) and then saturated (∼350 °C) C 3s, and finally CH_4_ (∼400–450 °C), as anticipated based on Figure 4. In the presence of O_2_, CO is oxidized at lower temperatures (slightly above 100 °C on LFC) compared to the simpler CO + NO mixture, as shown in Figure 3. Consistently, CO conversion approaches 100% at 300 °C (or below, for LFC), again significantly lower than the CO + NO mixture. While this lowering of the range of temperature to achieve the same activity might also be an effect of a longer contact time with the gases (i.e., lower WHSV, see Table 2), the ranking of activity in CO oxidation of the materials appears very different with the complex TWC mixture vs the simple CO + NO. Now the LFC appears to be markedly more active than LCFC, and the production route does not make a difference on the activity of the latter. With just CO and NO, the oxidation of CO was found to be more effective on LCFC prepared by COP, while both materials prepared by FSP (LFC and LCFC) behave similarly. Note that the reactant conversion on the same mass of the catalyst does not account for differences in the specific surface of the materials, see Table 1, where there is a factor of up to 3 among the measured values. The same measurements of Figure 5 reformulated through an estimate of turn-over frequency, thus accounting for the specific surface, is reported in the Supporting Information (Figure S3). They unambiguously suggest a specific activity ranking as LCFC-COP > LFC-FSP > LCFC-FSP (the reverse order of specific surface). NO reduction is negligible in the presence of stoichiometric O_2_; some activity (not reported in Figure 5) was observed between 350 and 500 °C, with a maximum conversion of 7 and 2.7% for FSP and COP LCFCs, respectively. Comparing the different catalysts, a large role of Ca doping, particularly in the oxidation of CO, C_3_H_6_, and CH_4_, is observed. The activation of methane is more affected by the synthesis, achieving 61% conversion at 600 °C for LCFC- FSP, whereas only 31% is converted for LCFC-COP. It also appears that the undoped ferrite LCF makes the ignition easier, specifically of CO and C_3_H_6_, suggesting that Ca doping is not effective. There is an abundance of O_2_ in the gas phase; it is extremely large at low temperatures, where oxidations did not start yet, but also at high temperatures some O_2_ remains, being difficult to complete the CH_4_ oxidation, see Figure 5.

NO reduction was not observed because of three facts: (i) the testing procedure, that fully oxidizes the materials with air at 600 °C; reagents are fed starting from this step, at 600 °C, where the catalysts are expected to lack oxygen vacancies; (ii) the abundance of O_2_ mentioned that increases during cooling, prevents the formation of enough vacancies; and (iii) adsorption of other gases might be preferred over the adsorption of NO on the perovskite surface.

The activity with the rich mixture is quite different, as shown in Figure 6. First, the evolution of CO, H_2_, and HC is the result of two mechanisms, oxidation and reforming, the latter emerging at high temperatures, where O_2_ is completely consumed in the first part of the catalyst bed. Second, the phase in which oxygen is completely consumed allows a significant (up to quantitative) conversion of NO. That is a confirmation of the results of the CO + NO mixture, where the mechanism of NO reduction based on vacancy formation was suggested, but it requires reaching the extinction of O_2_ in the gas phase to recreate vacancies, after the oxidative pretreatment of the catalysts at high temperatures. Indeed, NO reduction occurs above the temperature, where O_2_ is totally consumed. We did not observe any activation of CH_4_ oxidation (not shown). We clearly see that both CO and H_2_ are still available when O_2_ is totally consumed (>350 °C), likely supporting the NO reduction. The NO reduction is better on the COP sample compared to the FSP, supposedly sue to the larger amount of lattice oxygen identified by XPS for COP-prepared materials. Moreover, the two copper species detected by TPR seem to work consequently, with a first activation in CO conversion at 350 °C and a second rise in reactivity a 400–450 °C.

Between 200 and 350 °C, we observe the activation of CO, H_2_, and propene oxidation. C_3_H_8_ oxidation is surprisingly very weak at rich conditions, compared to the stoichiometric mixture, perhaps due to the different propane/oxygen ratio. A maximum in C_3_H_6_ conversion is reached when O_2_ in the gas feed approaches the total consumption, at about 380 °C. At higher temperatures (>400 °C), the production of CO and H_2_ conversion ceases to increase because of the activation of the reforming reactions that is especially clear for the COP sample. For temperatures higher than 500 °C, the CO and H_2_ conversion stabilizes, although O_2_ is not available anymore. This indicates that C_3_H_6_ consumption is correlated to the action of another oxidizing agent, such as NO. An interesting mechanism occurs for the sample LCFC—FSP; NO starts to convert at 290 °C for both LCFC catalysts, but in the case of the LCFC-FSP sample, the reaction seems to follow a two-step mechanism: the first at low temperature, with a maximum at 350 °C (7% conversion), whereas after the total O_2_ consumption, NO conversion increases, reaching the total reduction at 590 °C. The LCFC-COP sample does not exhibit such a behavior, neither does LFC. The comparison of catalysts with the rich TWC reformulated to account for the specific surface area is reported in the Supporting Information (Figure S4).

The results of the activity test with the complex TWC mixture for LFC FSP, very similar to LCFC-COP, confirm the hypothesis that LCFC-COP was not effectively doped with Ca. Comparing LFC FSP and LCFC FSP (i.e., the same composition with and without Ca doping), the undoped sample appears more active in NO reduction. For this reason, we can conclude that the inclusion of Ca has a negative impact on the catalytic reductive activity at these conditions, rearranging the perovskite lattice in such a way that compromises the reducibility of the transition metals (as seen by H_2_-TPR) with a particular reference to Cu. Comparing the XPS and EDX composition it is evident that LCFC COP is not the sample with the highest amount of Fe and Cu and thus, the surface composition does not appear to play a fundamental role under this reaction condition (i.e., from high to low temperature after pretreatment). In contrast, the possible formation of oxygen vacancies capable of activating HCs seems to play a fundamental role. Oxygen vacancies can be formed thanks mainly to the under stoichiometry and are observed not to have undergone cluster formation with Ca inserted into the unit cell.^34^

### 3.2 Variability in La_0.6_Ca_0.2_Fe_0.8_Cu_0.2_O_3_ Synthesis by FSP

#### 3.2.1 Characterization

An extensive description of the characterization results is reported in the Supporting Information. In general, these samples show significant differences in morphology, composition, and structural features. Ca insertion in the perovskite lattice differs with the production stage of collection of the sample. XP spectroscopy confirms an incomplete formation of the perovskite structure in the early stages of the production by FSP, with a wide contribution of amorphous (according to XRD analysis) La and Fe oxides. This is also suggested by the broadened shape of the La 3d XP spectrum, which is absent for the other samples, meaning that La(OH)_3_ species are more present in the first stages of the synthesis.^44−53,55,56,61^ The oxidation of the samples is also increasing going from A to E, as seen from the signal of Fe 2p and TPR curves.^3,4,62^ Oxygen species are present in the surface for each sample. From TPR analysis, the reducibility of the products of early stages is lower, probably due to a lower degree of crystallinity, as seen in similar systems in literature.^15,22,63^

Samples collected at similar operating conditions in the FSP setup show a similar behavior with regards to Fe(IV) reducibility.

The samples appear homogenous from SEM images and surface area determination (see Table 1).

#### 3.2.2 Catalytic Activity

**Figure 7:**
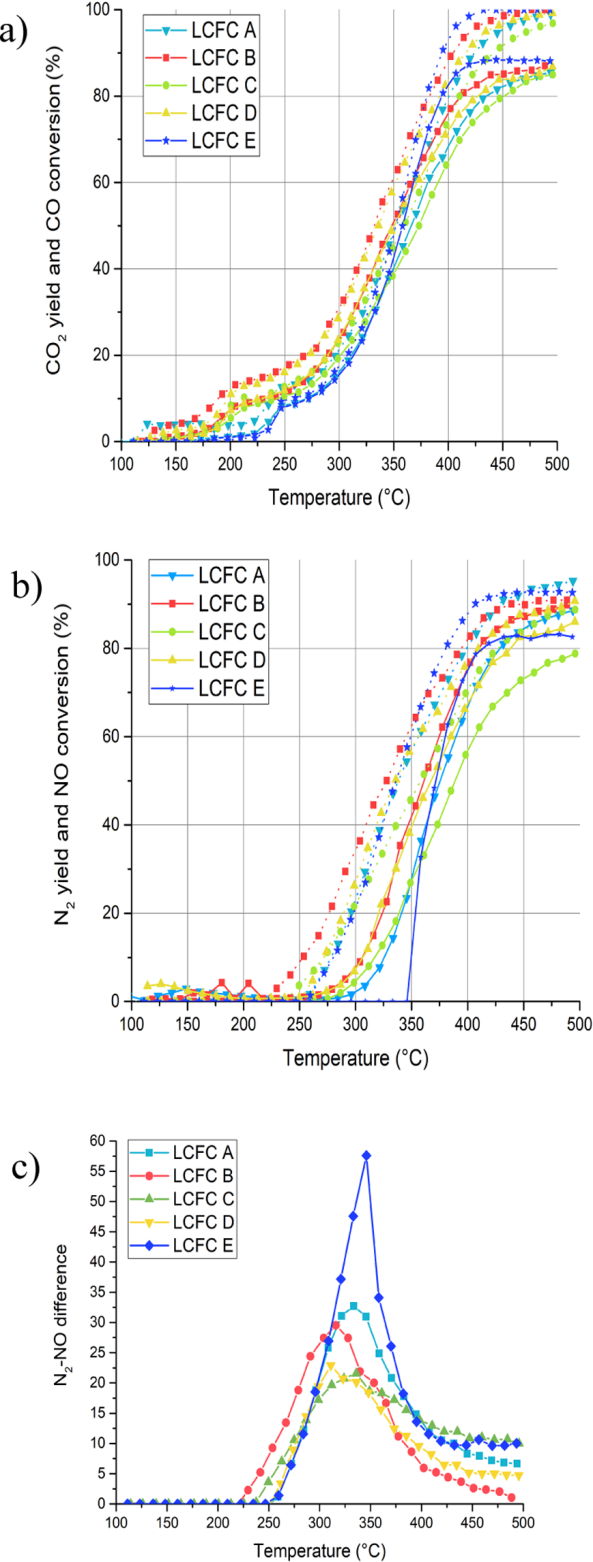
Activity during CO + NO reaction. Samples LCFC A, B, C, D, and E. (a,b) CO and NO conversion (dashed) and CO_2_ and N_2_ yields (solid) and (c) N_2_O determined by the difference between NO conversion and N_2_ yield.

Each sample was tested for CO-assisted NO reduction (Figure 7).

Samples A, C, and E have also been tested with a complex mixture (see Figures S8 and S9 in Supporting Information, respectively stoichiometric and rich mixture), given their remarkable differences in properties as revealed by the structural and compositional analyses.

The two steps in the CO conversion that we have seen in Figure 3 are confirmed, supporting the evidence of a Mars–van Krevelen mechanism at these conditions (no O_2_) and testing procedure (increasing temperature, from RT). The LCFC B and D are the more active catalysts, slightly better than A, C, and E. The conversions of both CO and NO are in excess of 80–85% above 450 °C. Samples B and D are characterized by the higher (Fe + Cu)/(La + Ca) atomic ratio (1.3 and 1.4, respectively—see Table S2 in Supporting Information). The efficiency of Ca insertion does not seem to enhance reactivity: the activity of catalysts B and D are very similar, whereas the XRD patterns suggest a different insertion. This supports the hypothesis that at lower temperatures the surface active sites are more relevant for catalytic performances than bulk mobility. TPR revealed that sample B is characterized by the lower Cu(II) reduction temperature (Figure 2). We observe a notable difference in the N_2_ yield of sample D, which was characterized by a different crystallographic feature, although the CO oxidizes almost like the other materials, that is consistent with a higher N_2_O production even at high temperatures.

Considering stoichiometric TWC feed mixture, as shown in Figure S8, sample C is clearly less effective than A and its replica E in CO (and partially in C_3_H_6_) oxidation, while A and E confirm a very good reproducibility of the synthetic process. In the Supporting Information a table of comparison with a commercially available compound is reported (Table S4).

That is further confirmed by the O_2_ consumption, a useful parameter to summarize the total activity of each catalyst; it defines which reactions take place and the environment in which the reactions occur. As already seen above, oxygen conversion is never complete; at the maximum temperature of the test (600 °C) it reaches 93%.

Accordingly, the materials are always in the presence of some oxygen. The apparent two phases indicated by the oxygen conversion curve reflect the early activation of CO and H_2_, quite separate from the activation of HCs. Apparently, propene oxidation begins after the complete consumption of CO is achieved (>350 °C), suggesting that the same active sites are used. At very high temperatures, the differences among samples disappear not only because some species (CO, C 3s) are totally consumed but also because the mild CH_4_ conversion is very similar for all the samples. Also, NO, which is poorly reduced in a stoichiometric mixture, does not differentiate the samples. It appears that a lower crystallinity, together with the residual presence of metal oxides (sample A), turns out to be helpful to enhance catalytic activity.

At rich conditions, as shown in Figure S9, we see similar activity in all the samples, regardless of the production phase. Now oxygen is the limiting reagent, mostly for low-temperature oxidations, when NO is not yet activated (to produce O_2_). All the samples catalytically activate the combustion at about 180 °C for CO, H_2_, and propene oxidation. After O_2_ total consumption, propene reforming reaction occurs at about 400 °C, causing an increase in the CO concentration (conversion decreases) very clear at high temperatures (>530 °C). NO is reduced easily at 290 °C, but only at a total O_2_ consumption does NO conversion boost. Again, the reaction apparently follows a two-step mechanism in the presence (300 °C < *T* < 400 °C) or absence (>400 °C) of O_2_, finally leading to NO total conversion. These observations agree with those of Barbero et al.^43^ that attributed the activity in ethanol oxidation to the Fe(IV) surface sites and that of hydrocarbons to the surface oxygen vacancies (not probable in the presence of oxygen).

While differences among samples collected at different production phases are small, and the reproducibility at the same production phase (A vs E) is excellent, we see some residual variability, with sample C slightly less effective in CO and NO, while samples A and E activate C_3_H_6_ at lower temperatures. Considering the characterization, the higher activity of sample A, richer in lattice oxygen than in surface oxygen species, suggests a bulk effect on the functional tests rather than a surface phenomenon.

The main differences between samples A and C is the lower amount of Fe + Cu and the very low inclusion of Ca in the unit cell observed for A. An explanation of the higher catalytic activity of A, which also support the idea of a higher contribution of the bulk to the reactivity, may be the decrease of oxygen ion mobility due to the inclusion of calcium into the perovskite lattice.

The larger presence of Fe(II) indicated by TPR should also be considered. The testing procedure, with a pretreatment in oxygen at high temperatures, may be responsible for its oxidation to Fe(III) and Fe(IV), more active in oxidation. That is consistent with the activity in the simple mixture and with the CO oxidation two-step mechanism. Further work is in course to better understand this hypothesis.

## 4 Conclusions

In this article, we tackle the problem of industrial production of perovskite-based complex catalysts focusing on the effect of preparation procedure and reproducibility. La_0.8_Ca_0.2_Fe_0.8_Cu_0.2_O_3_ (LCFC), a Ca, Cu-doped Fe-based perovskite, is industrially synthesized by means of two different methods: coprecipitation COP and FSP. FSP powders obtained at successive moments have been compared, as well as a Ca-free catalyst (LaFe_0.8_Cu_0.2_O_3_, LFC).

XRD patterns suggest that the Ca insertion depends on the synthesis procedure (being more efficient in FSP) and, focusing on FSP, on the specific production moment. XPS and EDX underline a different surface composition and different segregation phenomena for COP and FSP catalysts. In the COP catalyst, in fact, the amount of surface oxygen is lower but the B cations, particularly Cu, are more abundant. Morphological differences are highlighted by SEM; the COP sample is more compact.

Consistently with the different chemical, structural, and morphological characteristics, the catalysts exhibit different activity and selectivity. For these reasons, the composition of the bulk and surface are quite significant for the catalytic performance; this holds, in particular, for the concentration of active oxygen species, ability to undergo Mars–van Krevelen oxidation mechanism, and B cation distribution along the material depth. Interestingly, the doping of the A site deeply alters the mobility of ions throughout the structure, resulting in different and, in some cases, worse catalytic activity. Moreover, the effect of the surface area is not directly related to the catalytic activity at these conditions because the most active samples are the ones with the lowest surface area values, evidencing the paramount role of surface/bulk composition and reducibility. In particular, the surface composition seems to play a more significant role at lower temperatures, whereas at higher temperatures, the reducibility is more relevant. Therefore, it is important to test not only under simple gas mixtures (e.g., CO + NO) but also more complex mixtures at relevant conditions (e.g., stoich or rich), especially for industrial applications.

This comparison allowed us to better clarify the role of calcium doping in catalytic activity, which depends on the reactant mixture and the reaction conditions. As a general trend, the COP technique results in a less efficient inclusion of Ca in the perovskite lattice and seems less suitable for the industrial approach. The insertion of Ca into the perovskite crystalline cell enhances the formation of Fe(IV) active sites for molecule oxidation. The presence of Ca inside the lattice, however, also affects the perovskite lattice, stabilizing copper with respect to the segregation of Cu(II) as copper oxide. In general, this decreases the reducibility of the catalyst. Oxygen vacancies are observed to prevail with the amount of Ca doping being higher than the ones characterizing the samples presented in this contribution. Because of this reason, it is probable that they can contribute to the activation of HCs only at higher temperatures when oxygen is not present anymore in the reaction mixture, being consumed in the oxidation of non-HC species.

## Supporting Information Available

Experimental conditions during collection of the samples; extensive characterization results for LCFC A, B, C, D, and E; XP spectra of the sample LFC FSP, LCFC COP, LCFC A, B, C, D, and E (O 1s, La 3d, and Fe 2p); SEM images for LFC, LCFC FSP, and LCFC COP; LCFC FSP and COP catalytic activity in stoichiometric and rich mixtures; SEM images for LCFC A, B, C, and D; catalytic setup scheme for complex mixture testing; composition table with comparison between XPS and EDX results; ICP compositional results; and table to compare commercially available alternative catalysts, and catalytic activity of LCFC A, C, and D for the complex mixture (PDF)

## References

[ref1] Libby W. F. Promising Catalyst for Auto Exhaust. Science 1971, 171, 499–500. 10.1126/science.171.3970.499. 4099422

[ref2] Royer S.; Duprez D.; Can F.; Courtois X.; Batiot-Dupeyrat C.; Laassiri S.; Alamdari H. Perovskites as Substitutes of Noble Metals for Heterogeneous Catalysis: Dream or Reality. Chem. Rev. 2014, 114, 10292–10368. 10.1021/cr500032a. 25253387

[ref3] Sorenson S. C.; Wronkiewicz J. A.; Sis L. B.; Wirtz G. P. Properties of LaCoO3 as a Catalyst in Engine Exhaust Gases. Ceram. Bull. 1974, 53, 446–449.

[ref4] Glisenti A.; Pacella M.; Guiotto M.; Natile M. M.; Canu P. Largely Cu-Doped LaCo1-XCuxO3 Perovskites for TWC: Toward New PGM-Free Catalysts. Appl. Catal., B 2016, 180, 94–105. 10.1016/j.apcatb.2015.06.017.

[ref5] Koirala R.; Pratsinis S. E.; Baiker A. Synthesis of Catalytic Materials in Flames: Opportunities and Challenges. Chem. Soc. Rev. 2016, 45, 3053–3068. 10.1039/c5cs00011d. 27108487

[ref6] Peña M. A.; Fierro J. L. G. Chemical Structures and Performance of Perovskite Oxides. Chem. Rev. 2001, 101, 1981–2018. 10.1021/cr980129f. 11710238

[ref7] Schwarz J. A.; Contescu C.; Contescu A. Methods for Preparation of Catalytic Materials. Chem. Rev. 1995, 95, 477–510. 10.1021/cr00035a002.

[ref8] Szabo V.; Bassir M.; Van Neste A.; Kaliaguine S. Perovskite-type oxides synthesized by reactive grinding. Appl. Catal., B 2002, 37, 175–180. 10.1016/s0926-3373(01)00328-9.

[ref9] Li Z.; Lv L.; Ao X.; Li J.-G.; Sun H.; An P.; Xue X.; Li Y.; Liu M.; Wang C.; Liu M. An Effective Method for Enhancing Oxygen Evolution Kinetics of LaMO3 (M=Ni, Co, Mn) Perovskite Catalysts and Its Application to a Rechargeable Zinc–Air Battery. Appl. Catal., B 2020, 262, 118291 10.1016/j.apcatb.2019.118291.

[ref10] Levasseur B.; Kaliaguine S. Effects of Iron and Cerium in La1-YCeyCo1-XFexO3 Perovskites as Catalysts for VOC Oxidation. Appl. Catal., B 2009, 88, 305–314. 10.1016/j.apcatb.2008.11.007.

[ref11] Fang F.; Feng N.; Wang L.; Meng J.; Liu G.; Zhao P.; Gao P.; Ding J.; Wan H.; Guan G. Fabrication of Perovskite-Type Macro/Mesoporous La1-XKxFeO3-Δ Nanotubes as an Efficient Catalyst for Soot Combustion. Appl. Catal., B 2018, 236, 184–194. 10.1016/j.apcatb.2018.05.030.

[ref12] Giannakas A. E.; Ladavos A. K.; Pomonis P. J. Preparation, Characterization and Investigation of Catalytic Activity for NO+CO Reaction of LaMnO3 and LaFeO3 Perovskites Prepared via Microemulsion Method. Appl. Catal., B 2004, 49, 147–158. 10.1016/j.apcatb.2003.12.002.

[ref13] Safakas A.; Bampos G.; Bebelis S. Oxygen Reduction Reaction on La0.8Sr0.2CoxFe1-XO3-Δ Perovskite/Carbon Black Electrocatalysts in Alkaline Medium. Appl. Catal., B 2019, 244, 225–232. 10.1016/j.apcatb.2018.11.015.

[ref14] Brewster J. H.; Kodas T. T. Generation of Unagglomerated, Dense, BaTiO3 Particles by Flame-Spray Pyrolysis. AIChE J. 1997, 43, 2665–2669. 10.1002/aic.690431310.

[ref15] Leanza R.; Rossetti I.; Fabbrini L.; Oliva C.; Forni L. Perovskite catalysts for the catalytic flameless combustion of methane. Appl. Catal., B 2000, 28, 55–64. 10.1016/s0926-3373(00)00163-6.

[ref16] Petrović S.; Terlecki-Baričević A.; Karanović L.; Kirilov-Stefanov P.; Zdujić M.; Dondur V.; Paneva D.; Mitov I.; Rakić V. LaMO3 (M = Mg, Ti, Fe) Perovskite Type Oxides: Preparation, Characterization and Catalytic Properties in Methane Deep Oxidation. Appl. Catal., B 2008, 79, 186–198. 10.1016/j.apcatb.2007.10.022.

[ref17] Faye J.; Baylet A.; Trentesaux M.; Royer S.; Dumeignil F.; Duprez D.; Valange S.; Tatibouët J.-M. Influence of Lanthanum Stoichiometry in La 1-XFeO 3-δ Perovskites on Their Structure and Catalytic Performance in CH 4 Total Oxidation. Appl. Catal., B 2012, 126, 134–143. 10.1016/j.apcatb.2012.07.001.

[ref18] Wang H.; Dong X.; Zhao T.; Yu H.; Li M. Dry Reforming of Methane over Bimetallic Ni-Co Catalyst Prepared from La(CoxNi1-x)0.5Fe0.5O3 Perovskite Precursor: Catalytic Activity and Coking Resistance. Appl. Catal., B 2019, 245, 302–313. 10.1016/j.apcatb.2018.12.072.

[ref19] Garcia-Muñoz P.; Lefevre C.; Robert D.; Keller N. Ti-Substituted LaFeO3 Perovskite as Photoassisted CWPO Catalyst for Water Treatment. Appl. Catal., B 2019, 248, 120–128. 10.1016/j.apcatb.2019.02.030.

[ref20] Pang S.; Xu J.; Su Y.; Yang G.; Zhu M.; Cui M.; Shen X.; Chen C. The Role of A-Site Cation Size Mismatch in Tune the Catalytic Activity and Durability of Double Perovskite Oxides. Appl. Catal., B 2020, 270, 118868 10.1016/j.apcatb.2020.118868.

[ref21] Tian C.; Zhang H.; Zhu X.; Lin B.; Liu X.; Chen H.; Zhang Y.; Mullins D. R.; Abney C. W.; Shakouri M.; Chernikov R.; Hu Y.; Polo-Garzon F.; Wu Z.; Fung V.; Jiang D.-e.; Liu X.; Chi M.; Liu Jimmy J.; Dai S. A New Trick for an Old Support: Stabilizing Gold Single Atoms on LaFeO3 Perovskite. Appl. Catal., B 2020, 261, 118178 10.1016/j.apcatb.2019.118178.

[ref22] Taran O. P.; Ayusheev A. B.; Ogorodnikova O. L.; Prosvirin I. P.; Isupova L. A.; Parmon V. N. Perovskite-like Catalysts LaBO3 (B=Cu, Fe, Mn, Co, Ni) for Wet Peroxide Oxidation of Phenol. Appl. Catal., B 2016, 180, 86–93. 10.1016/j.apcatb.2015.05.055.

[ref23] Grabowska E. Selected Perovskite Oxides: Characterization, Preparation and Photocatalytic Properties-A Review. Appl. Catal., B 2016, 186, 97–126. 10.1016/j.apcatb.2015.12.035.

[ref24] Li P.; Zhang R.; Liu N.; Royer S. Efficiency of Cu and Pd Substitution in Fe-Based Perovskites to Promote N2 Formation during NH3 Selective Catalytic Oxidation (NH3-SCO). Appl. Catal., B 2017, 203, 174–188. 10.1016/j.apcatb.2016.10.021.

[ref25] Zhang Q.; Huang Y.; Peng S.; Zhang Y.; Shen Z.; Cao J.-j.; Ho W.; Lee S. C.; Pui D. Y. H. Perovskite LaFeO3-SrTiO3 Composite for Synergistically Enhanced NO Removal under Visible Light Excitation. Appl. Catal., B 2017, 204, 346–357. 10.1016/j.apcatb.2016.11.052.

[ref26] Afzal S.; Quan X.; Zhang J. High Surface Area Mesoporous Nanocast LaMO3 (M = Mn, Fe) Perovskites for Efficient Catalytic Ozonation and an Insight into Probable Catalytic Mechanism. Appl. Catal., B 2017, 206, 692–703. 10.1016/j.apcatb.2017.01.072.

[ref27] Wang M.; Zhao T.; Dong X.; Li M.; Wang H. Effects of Ce Substitution at the A-Site of LaNi0.5Fe0.5O3 Perovskite on the Enhanced Catalytic Activity for Dry Reforming of Methane. Appl. Catal., B 2018, 224, 214–221. 10.1016/j.apcatb.2017.10.022.

[ref28] Gong S.; Xie Z.; Li W.; Wu X.; Han N.; Chen Y. Highly Active and Humidity Resistive Perovskite LaFeO3 Based Catalysts for Efficient Ozone Decomposition. Appl. Catal., B 2019, 241, 578–587. 10.1016/j.apcatb.2018.09.041.

[ref29] Sun W.; Wei H.; yang An L.; Jin C.; Wu H.; Xiong Z.-a.; Pu C.; Sun C. Oxygen Vacancy Mediated La1-XCexFeO3-Δ Perovskite Oxides as Efficient Catalysts for CWAO of Acrylic Acid by A-Site Ce Doping. Appl. Catal., B 2019, 245, 20–28. 10.1016/j.apcatb.2018.12.024.

[ref30] Wu J.; Dacquin J. P.; Cordier C.; Dujardin C.; Granger P. Optimization of the Composition of Perovskite Type Materials for Further Elaboration of Four-Way Catalysts for Gasoline Engine. Top. Catal. 2019, 62, 368–375. 10.1007/s11244-018-1083-2.

[ref31] Emmerlich J.; Linke B. M.; Music D.; Schneider J. M. Towards Designing La1 - XSrxCoyFe 1 - YO3 - D with Enhanced Phase Stability: Role of the Defect Structure. Solid State Ionics 2014, 255, 108–112. 10.1016/j.ssi.2013.11.047.

[ref32] Schön A.; Dacquin J.-P.; Granger P.; Dujardin C. Non Stoichiometric La1-YFeO3 Perovskite-Based Catalysts as Alternative to Commercial Three-Way-Catalysts? – Impact of Cu and Rh Doping. Appl. Catal., B 2018, 223, 167–176. 10.1016/j.apcatb.2017.06.026.

[ref33] Wu Y.; Ni X.; Beaurain A.; Dujardin C.; Granger P. Stoichiometric and Non-Stoichiometric Perovskite-Based Catalysts: Consequences on Surface Properties and on Catalytic Performances in the Decomposition of N 2O from Nitric Acid Plants. Appl. Catal., B 2012, 125, 149–157. 10.1016/j.apcatb.2012.05.033.

[ref34] Pushpa R.; Daniel D.; Butt D. P. Electronic Properties of Ca Doped LaFeO3: A First-Principles Study. Solid State Ionics 2013, 249–250, 184–190. 10.1016/j.ssi.2013.08.007.

[ref35] Ciambelli P.; Cimino S.; Lisi L.; Faticanti M.; Minelli G.; Pettiti I.; Porta P. La, Ca and Fe Oxide Perovskites: Preparation, Characterization and Catalytic Properties for Methane Combustion. Appl. Catal., B 2001, 33, 193–203. 10.1016/s0926-3373(01)00163-1.

[ref36] Shirley D. A. High-Resolution x-Ray Photoemission Spectrum of the Valence Bands of Gold. Phys. Rev. B: Solid State 1972, 5, 4709–4714. 10.1103/physrevb.5.4709.

[ref37] Briggs D. Handbook of X-Ray Photoelectron Spectroscopy C. D. Wanger, W. M. Riggs, L. E. Davis, J. F. Moulder and G. E.Muilenberg Perkin-Elmer Corp., Physical Electronics Division, Eden Prairie, Minnesota, USA, 1979. 190 pp. $195; Chastain J., Ed.; Wiley Analytical Science: Eden Prairie, 1981; Vol. 3.

[ref38] Umbach E. Practical Surface Analysis; Wiley: New York, 1992; Vol. 11.

[ref39] Garbujo A.; Pacella M.; Natile M. M.; Guiotto M.; Fabro J.; Canu P.; Glisenti A. On A-Doping Strategy for Tuning the TWC Catalytic Performance of Perovskite Based Catalysts. Appl. Catal., A 2017, 544, 94–107. 10.1016/j.apcata.2017.07.009.

[ref40] Pecchi G.; Jiliberto M. G.; Buljan A.; Delgado E. J. Relation between Defects and Catalytic Activity of Calcium Doped LaFeO 3 Perovskite. Solid State Ionics 2011, 187, 27–32. 10.1016/j.ssi.2011.02.014.

[ref41] Galenda A.; Natile M. M.; Nodari L.; Glisenti A. La0.8Sr0.2Ga0.8Fe0.2O3-δ: Influence of the Preparation Procedure on Reactivity toward Methanol and Ethanol. Appl. Catal., B 2010, 97, 307–322. 10.1016/j.apcatb.2010.04.004.

[ref42] Shannon R. D. Revised Effective Ionic Radii and Systematic Studies of Interatomic Distances in Halides and Chalcogenides. Acta Crystallogr., Sect. A: Cryst. Phys., Diffr., Theor. Gen. Crystallogr. 1976, 32, 751–767. 10.1107/s0567739476001551.

[ref43] Barbero B. P.; Gamboa J. A.; Cadús L. E. Synthesis and Characterisation of La1-XCaxFeO3 Perovskite-Type Oxide Catalysts for Total Oxidation of Volatile Organic Compounds. Appl. Catal., B 2006, 65, 21–30. 10.1016/j.apcatb.2005.11.018.

[ref44] Tabata K.; Matsumoto I.; Kohiki S. Surface Characterization and Catalytic Properties of La1-x-SrxCoO3. J. Mater. Sci. 1987, 22, 1882–1886. 10.1007/bf01132422.

[ref45] Tejuca L. G.; Fierro J. L. G. XPS and TPD Probe Techniques for the Study of LaNiO3 Perovskite Oxide. Thermochim. Acta 1989, 147, 361–375. 10.1016/0040-6031(89)85191-3.

[ref46] Zhi-jian K.; Li-ping L.; Quan W. An XPS Study of Perovskite Oxides RECrO3. Chem. Res. Chin. Univ. 1996, 12, 280–284.

[ref47] Haber J.; Stoch J.; Ungier L. X-Ray Photoelectron Spectra of Oxygen in Oxides of Co, Ni, Fe and Zn. J. Electron Spectrosc. Relat. Phenom. 1976, 9, 459–467. 10.1016/0368-2048(76)80064-3.

[ref48] Mullica D. F.; Perkins H. O.; Lok C. K. C.; Young V. The X-Ray Photoemission Spectra of La(OH)3. J. Electron Spectrosc. Relat. Phenom. 1993, 61, 337–355. 10.1016/0368-2048(93)80024-g.

[ref49] Sunding M. F.; Hadidi K.; Diplas S.; Løvvik O. M.; Norby T. E.; Gunnæs A. E. XPS Characterisation of in Situ Treated Lanthanum Oxide and Hydroxide Using Tailored Charge Referencing and Peak Fitting Procedures. J. Electron Spectrosc. Relat. Phenom. 2011, 184, 399–409. 10.1016/j.elspec.2011.04.002.

[ref50] Naumkin V. A.; Kraut-Vass A.; Gaarenstroom S. W. NIST X-Ray Photoelectron Spectroscopy Database. Meas. Serv. Div. Natl. Inst. Stand. Technol. 2012, 20899, 20899 10.18434/T4T88K.

[ref51] Natile M. M.; Poletto F.; Galenda A.; Glisenti A.; Montini T.; Rogatis L. D.; Fornasiero P. La0.6Sr0.4Co1-YFeyO 3-δ Perovskites: Influence of the Co/Fe Atomic Ratio on Properties and Catalytic Activity toward Alcohol Steam-Reforming. Chem. Mater. 2008, 20, 2314–2327. 10.1021/cm703329k.

[ref52] Galenda A.; Natile M. M.; Krishnan V.; Bertagnolli H.; Glisenti A. LaSrCoFeO and Fe 2 O 3 /LaSrCoFeO Powders: Synthesis and Characterization. Chem. Mater. 2007, 19, 2796–2808. 10.1021/cm062742i.

[ref53] Natile M. M.; Ponzoni A.; Concina I.; Glisenti A. Chemical Tuning versus Microstructure Features in Solid-State Gas Sensors: LaFe1-XGaxO3, a Case Study. Chem. Mater. 2014, 26, 1505–1513. 10.1021/cm4018858.

[ref54] Guiotto M.; Pacella M.; Perin G.; Iovino A.; Michelon N.; Natile M. M.; Glisenti A.; Canu P. Washcoating vs. Direct Synthesis of LaCoO3 on Monoliths for Environmental Applications. Appl. Catal., A 2015, 499, 146–157. 10.1016/j.apcata.2015.04.013.

[ref55] Gunasekaran N.; Rajadurai S.; Carberry J. J.; Bakshi N.; Alcock C. B. Surface Characterization and Catalytic Properties of La1-XAxMO3 Perovskite Type Oxides. Part I. Studies on La0.95Ba0.05MO3 (M = Mn, Fe or Co) Oxides. Solid State Ionics 1994, 73, 289–295. 10.1016/0167-2738(94)90046-9.

[ref56] Perin G.; Fabro J.; Guiotto M.; Xin Q.; Natile M. M.; Cool P.; Canu P.; Glisenti A. Cu@LaNiO3 Based Nanocomposites in TWC Applications. Appl. Catal., B 2017, 209, 214–227. 10.1016/j.apcatb.2017.02.064.

[ref57] Andoulsi R.; Horchani-Naifer K.; Férid M. Structural and Electrical Properties of Calcium Substituted Lanthanum Ferrite Powders. Powder Technol. 2012, 230, 183–187. 10.1016/j.powtec.2012.07.026.

[ref58] Pillai S. K.; Sikhwivhilu L. M.; Hillie T. K. Synthesis, Characterization and Photoluminescence Properties of Dy3+-Doped Nano-Crystalline SnO2. Mater. Chem. Phys. 2010, 120, 619–624. 10.1016/j.matchemphys.2009.12.010.

[ref59] Mars P.; van Krevelen D. W. Oxidations Carried out by Means of Vanadium Oxide Catalysts. Chem. Eng. Sci. 1954, 3, 41–59. 10.1016/s0009-2509(54)80005-4.

[ref61] Pacella M.; Garbujo A.; Fabro J.; Guiotto M.; Xin Q.; Natile M. M.; Canu P.; Cool P.; Glisenti A. PGM-Free CuO/LaCoO3 Nanocomposites: New Opportunities for TWC Application. Appl. Catal., B 2018, 227, 446–458. 10.1016/j.apcatb.2018.01.053.

[ref62] McIntyre N. S.; Zetaruk D. G. X-Ray Photoelectron Spectroscopic Studies of Iron Oxides. Anal. Chem. 1977, 49, 1521–1529. 10.1021/ac50019a016.

[ref63] Rossetti I.; Biffi C.; Forni L. Oxygen Non-Stoichiometry in Perovskitic Catalysts: Impact on Activity for the Flameless Combustion of Methane. Chem. Eng. J. 2010, 162, 768–775. 10.1016/j.cej.2010.06.003.

