# Supplementary material for: Industrially Produced Fe- and Mn-Based Perovskites: Effect of Synthesis on Reactivity in Three-Way Catalysis: Part 1
Source: ACS Omega. 2021 Sep 14;6(38):24325–37. doi: 10.1021/acsomega.1c02133 (PMC8482407; doi:10.1021/acsomega.1c02133)
Supplement: Supplementary file 1 — ao1c02133_si_001.pdf [file ao1c02133_si_001.pdf]

Industrially Produced Fe- and Mn-based perovskites: Effect of Synthesis on Reactivity in Three-Way Catalysis. Part 1

Elena Brusamarello[a], Cataldo Blonda[d], Cristina Salazar-Castro[b], Andrea Eva Pascui[c], Paolo Canu [d], Antonella Glisenti [a,e]*

[a] Dept. of Chemical Sciences, University of Padova, - Via F. Marzolo, 1, 35131, Padova, Italy.

[b] L'Urederra Foundation, Perguita Industrial Area, No. 1 street, CP: 31210 Los Arcos (Navarra) Spain

[c] Johnson Matthey Technology Centre, Blount's Court Sonning Common, RG4 9NH, United Kingdom

[d] Dept. of Industrial Engineering, University of Padova, - Via F. Marzolo, 9, 35131, Padova, Italy.

[e] CNR-ICMATE, INSTM - Via F. Marzolo, 1, 35131, Padova, Italy.

Supplementary material

LCFC A, B, C, D and E: experimental conditions during collection

Being an industrial machinery the detailed knowledge of the synthesis parameters (Temperature, Pressure) is not possible but the main differences among the batches are summarized as follows. LCFC A is collected after saturation of filters, following an initial tuning of the process, in terms of temperature and pressure at the nozzle. LCFC B is collected on the filters after a following stable operation step, at the same conditions as case A; the temperature on the filters (where particles are collected) and ducts may rise, but it is always below 200°C; even if the residence time of the material is longer, we do not expect this temperature to affect the material composition or structure. After phase B, phase C attempted to stabilize the operating conditions in the flame to approach case A, with stable operation. The sample LCFC D is the last material extracted from the equipment and LCFC E is a duplicate sample of LCFC A. Each material (A, B, C, D, and E) is sampled in the same way: the nanopowders are retained as aggregates on the filters tissue and periodically discharged (by shaking the bag filters with compressed air) into a container, that is emptied before each phase, to limit cross-contamination. The collection of materials D and E, at the final stage, requires mechanical abrasion of the filters, because of a more compact deposition on the tissue.

LCFC A, B, C, D and E: H_2-TPR results

	T max (°C)	mol H_2 consumed/expected	Assignment	Fe(IV)/((Fe(IV)+Fe(III))
**LCFC FSP A**				0.27
Low T peak	290	0.68	Cu(II) - Cu (0)	
Broad signal	450-650	0.04	Fe(IV)-Fe(III); Fe(III)-Fe(II)	
**LCFC FSP B**				0.55
Low T peak	234	0.83	Cu(II) - Cu (0)	
Broad signal	450-650	0.09	Fe(IV)-Fe(III); Fe(III)-Fe(II)	
**LCFC FSP C**				0.13
Low T peak	252	0.89	Cu(II) - Cu (0)	
Broad signal	450-650	0.03	Fe(IV)-Fe(III); Fe(III)-Fe(II)	
**LCFC FSP D**				0.54
Low T peak	253	0.78	Cu(II) - Cu (0)	
Broad signal	450-650	0.05	Fe(IV)-Fe(III); Fe(III)-Fe(II)	
**LCFC FSP E**				0.47
Low T peak	272	0.65	Cu(II)-Cu(0)	
Broad signal	450-650	0.02	Fe(IV)-Fe(III); Fe(III)-Fe(II)	

Table S1. H_2-TPR results. The third column (mol H_2 consumed/expected) refers to the experimental and theoretical amount of H_2 to be consumed during the TPR, in correlation to the stoichiometric composition of the samples.

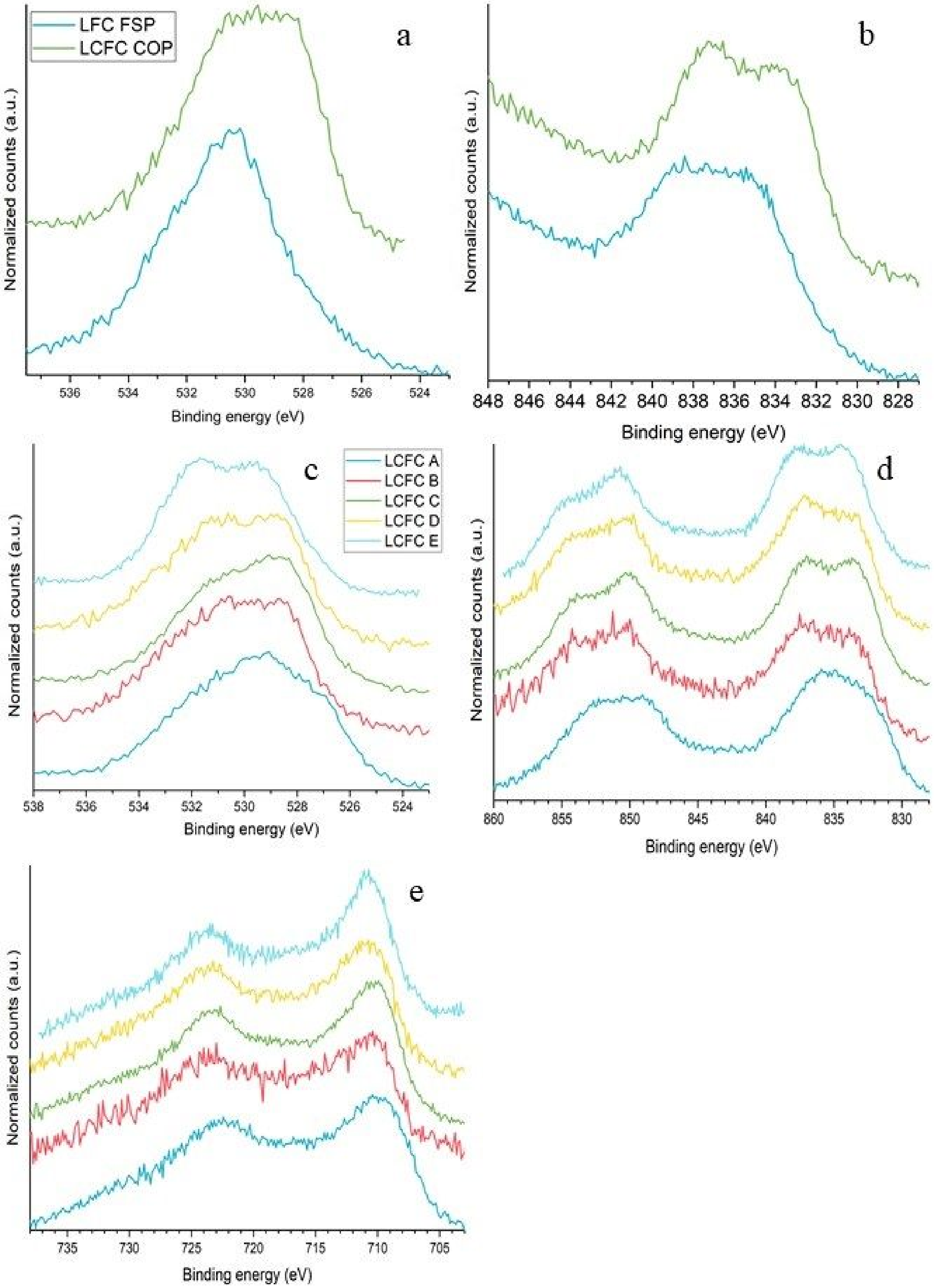

Figure S1. XP spectra of the samples LFC FSP and LCFC COP. a) O1s, b) La3d. XP spectra of the samples LCFC A, B, C, D and E. c) O1s, d) La3d, e)Fe2p

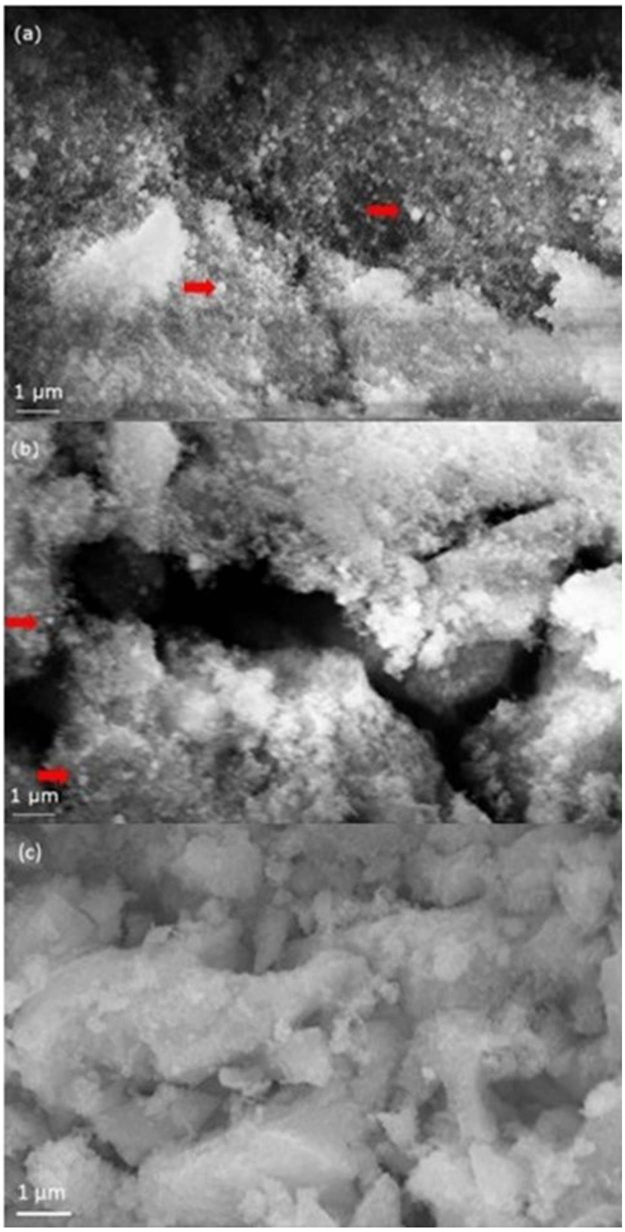

Figure S2. SEM images for LFC sample (a), LCFC FSP (b), LCFC COP (c).

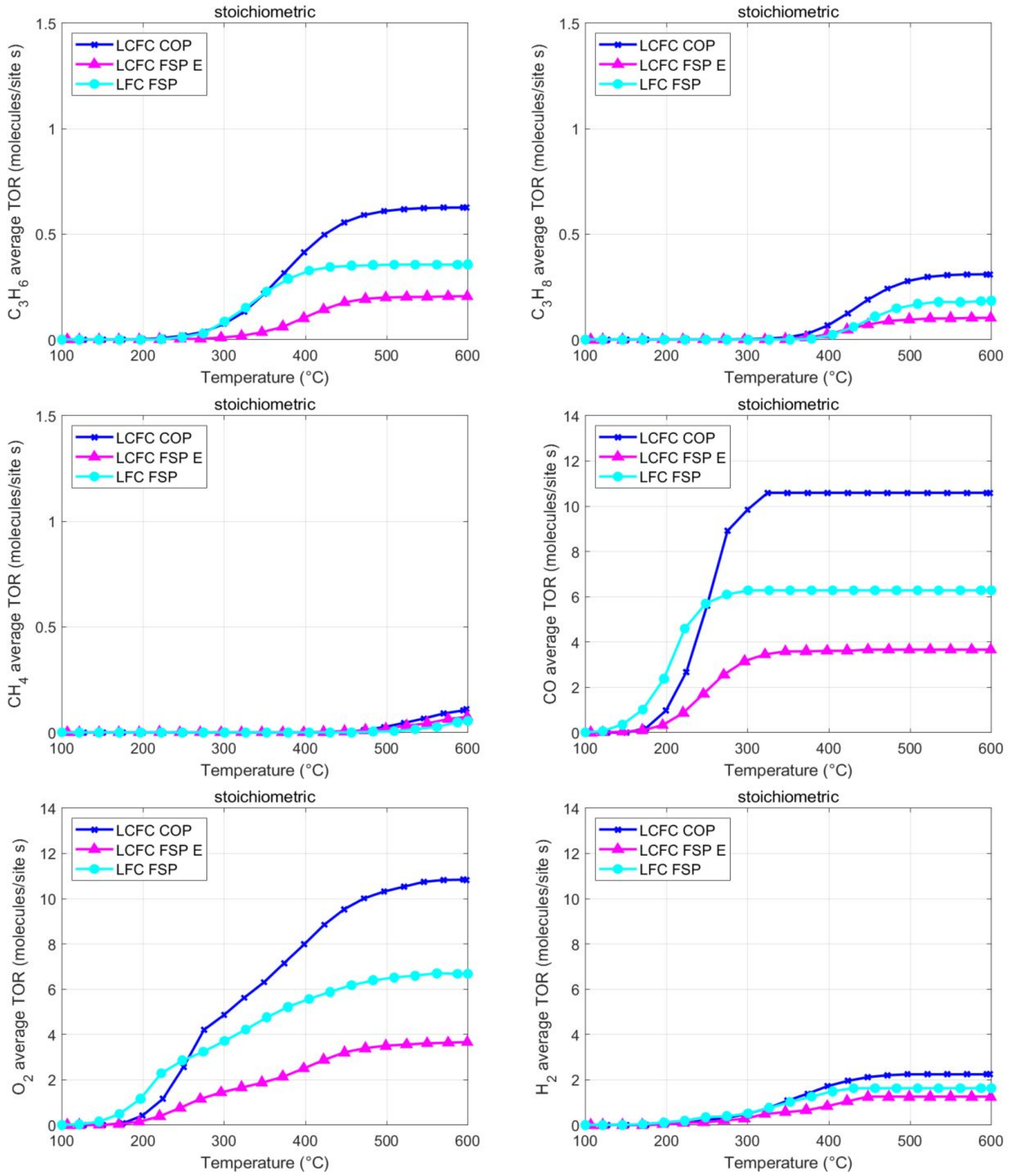

Figure S3: LCFC FSP, LCFC COP. Stoichiometric TWC mixture

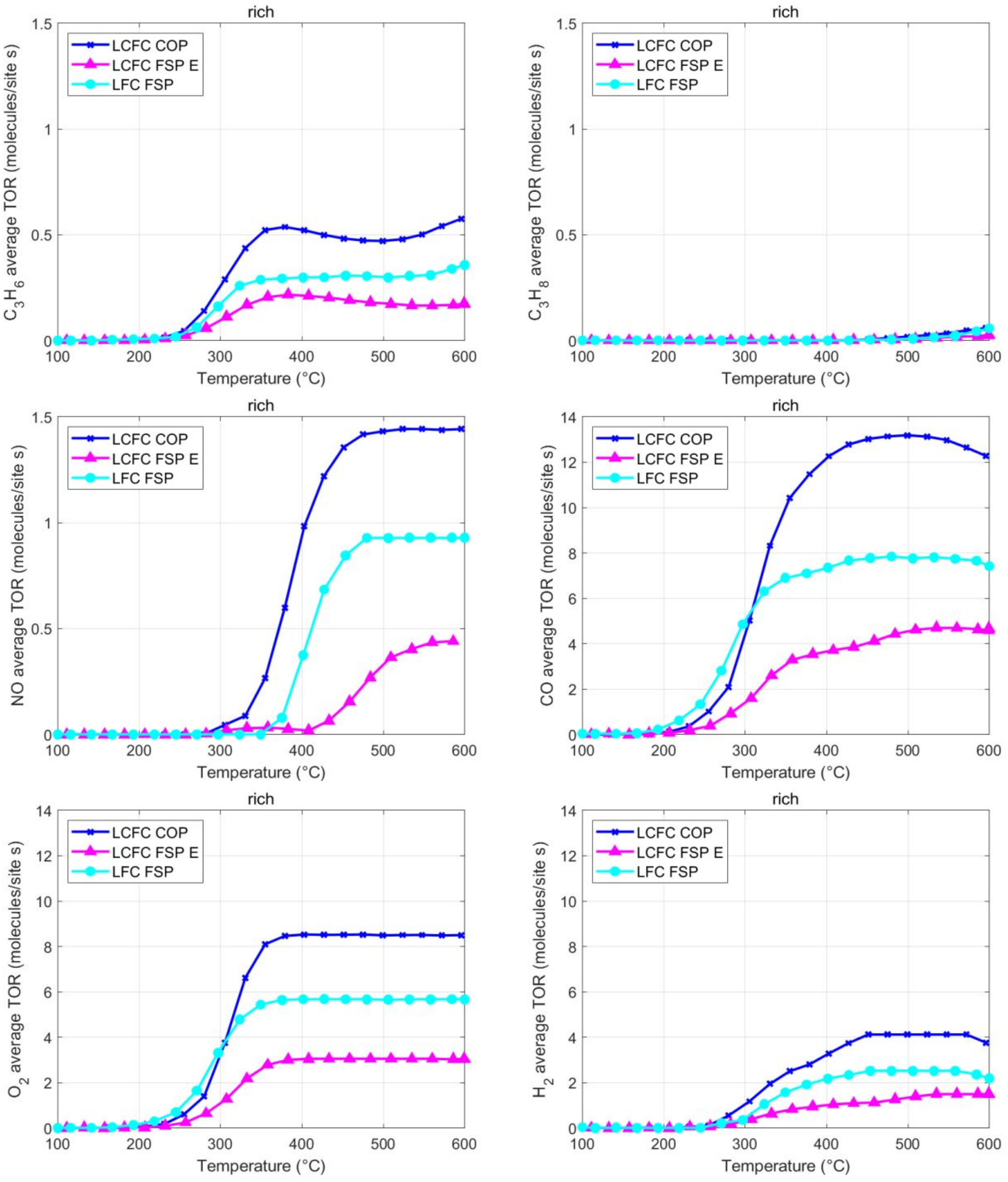

Figure S4: Activity of LFC FSP, LCFC FSP, LCFC COP. Rich TWC mixture.

LCFC A, B, C, D and E: Characterization

Five $La_{0.6}Ca_{0.2}Fe_{0.8}Cu_{0.2}O_3$ samples (LCFC A, B, C, D and E respectively) have been collected at different flame and plant operating conditions in the continuous FSP process. These samples show significant differences in morphology, composition and structural features. LCFC A diffraction pattern (Figure S5), unlike B, C and D, shows a lower degree of crystallinity, suggesting incomplete formation of the perovskite phase. In LCFC B, C, and E, the most relevant diffraction peak shifts from 32.25° to higher values (32.3°-32.5°) suggesting the change in the unit cell size due to the different radius of the cations[1] and confirms the incorporation of A-site dopants into the perovskite unit cell. Sample, D, in contrast, shows a decrease in the 2θ value of the peak at 32° consistent with an inefficient insertion of Ca.

XP spectra obtained for the different samples are compared in Figure S1 while atomic compositions (XPS and EDX) are summarized in Table S2. The O1s XPS signal has two components perovskite lattice (528.7 eV) and surface oxygen species, e.g. OH^-(530.8 eV).A difference is observed only in LCFC A in which a component centered at 529.0 eV is relevant; the peak position corresponds to oxygen in La_2O_3 and Fe_2O_3. [2] The peak shape indicates a high degree of hydroxylation of the surfaces.

O1s peak confirms that the first stage of the synthesis does not allow the complete formation of the perovskite structure, but instead an additional mixture of La and Fe oxides (contribution at about 529.5-530 eV), likely giving a contribution to the amorphous feature of the XR diffractograms of the initial samples.

The change of the O1s peak shape is evident as we move from sample A to E: in sample D lattice and hydroxyl oxygen are in comparable amounts. The La3d XP spectra are quite similar for all samples, and agree (peak position 832.8-833.2 eV and shake-up contribution at 835.9-837.5 eV) with lanthanum (III) in perovskites. The broadened shape of $La3d_{5/2}$ peak in sample A suggests the presence of another contribution, assigned to $La(OH)_3$/LaOOH species (834.5 eV) [see reference in the manuscript]. This last contribution is less evident in the samples B, C, and D.

The Fe2p XP spectra are significant, as they show a progressive shift towards higher BE going from sample A to E. $Fe2p_{3/2}$ peak centered at 709.4 eV in sample LCFC A, is typical of Fe(II). For sample D the peak position tends to reach the value of 710.2-710.5 eV, typical of $Fe2p_{3/2}$ in the Fe(III) form [3]. A further confirmation comes from the deconvolution of the spectra in the region of Fe2p (carried out setting the typical values for Fe(II) and Fe(III) reported in ref. [4]): the Fe(II)/Fe(III) atomic ratio being 1.8 in sample A and ca. zero in the other ones.

Different oxidation states for iron are also confirmed by TPR analysis, as reported below. Fe(IV), suggested by TPR, cannot be confirmed by XPS.[5]

Quantitative analysis (Table S1) shows that oxygen is always over stoichiometric confirming the presence of surface oxygen species. Iron is also relevant in surface even if it is slightly more abundant under the few external monolayers of samples A and C. Ca is near the nominal value only with EDX whereas Cu is more abundant in surface but always under-stoichiometric.

Cu2p XP spectra do not show any change in the oxidation state of copper, Cu(II). Ca2p XP spectra do not give any further insight about the surface structure and composition of the samples, this being quite similar for all samples and corresponding to the expected Ca(II).

Furthermore, EDX/XPS cation-only compositional analysis point out a trend of marked segregation of La on the surface, probably as oxide, whereas Fe surface segregates only in LCFC D. The B/A cations atomic ratio is near the expected value (being slightly higher only in the surface of sample B and D).

The bulk reducibility of the solids was investigated by H_2-Temperature Programmed Reduction experiments (H_2-TPR). TPR profile of the different LCFC (Figure 2 in the manuscript, Table S1) samples, A-E, shows the presence of two, in some cases three, signals at 224-286°C, 465°C and 582°C. The peak at lower temperature is ascribed to the reduction from Cu(II) to Cu(0) as reported in literature.[4] This assignment is in good agreement with the calculated hydrogen consumption (Table S1). The Cu(II) reduction temperature shifts throughout the different samples; the highest temperature is observed for sample A, which contains, beside the desired perovskite, a mixture of metal oxides (as shown by XPS and EDX). We can rationalize the different reduction temperature as the synthetic process conditions give rise to catalysts characterized by slightly different size and degree of crystallinity. In analogous samples obtained by FSP, Rossetti et al. [6] reported that highly reducible B^{3+} is evident for samples prepared with lower pressure drop across the nozzle, suggesting that a slightly higher crystal order induces higher reducibility. Furthermore, a better ordering leads to more energetically uniform redox species. This can be compared with Isupova et al. [7] who found that the tuning of B-O interaction strength correlates with the concentration of phase boundaries, and a higher reducibility is expected with increasing the concentration of phase boundaries. So, beside the inclusion of dopants, the reducibility of the sample depends also on the FSP synthetic conditions which may alter the morphological and crystalline character of the obtained materials.

The peak at higher temperature behaves differently: in some cases, two different contributions are clearly recognizable (e.g. LCFC A), in other cases they are merged into one single broad peak. The higher temperature region is attributed to iron species, in particular the reduction from Fe(IV) to Fe (III) (465 °C) and Fe(III) to Fe(II) (582 °C) [8,9].

The results of the deconvolution are summarized by the ratio of the integrated areas of the corresponding peaks in Table S1.

Samples B and D have a similar ratio Fe(IV)/Fe(III), compared to samples A, C and E which show almost no reduction of Fe(IV). Note that samples A and C were collected at similar operating conditions in the FSP set up.

SEM images (Figure S6) point out the homogeneity of the samples and underline the formation of highly dispersed particles and, progressively with the production process advancement, also of globular particles (diameter of about 100 nm), most likely combustion residues (carbon). For instance, sample A does not show any combustion residue, whereas the other samples progressively have more carbon particles on their surface.

The specific surface areas obtained for this samples are reported in Table 1 (in the manuscript): no significant differences are observed for the specific surface area of the catalysts obtained at different stages of the FSP process that range from 57 to 61 m^2/g testifying to the morphological homogeneity of the catalysts. In fact, focusing on FSP, the main influence seems to derive from the doping (LFC FSP specific surface area is about 32 m^2/g).

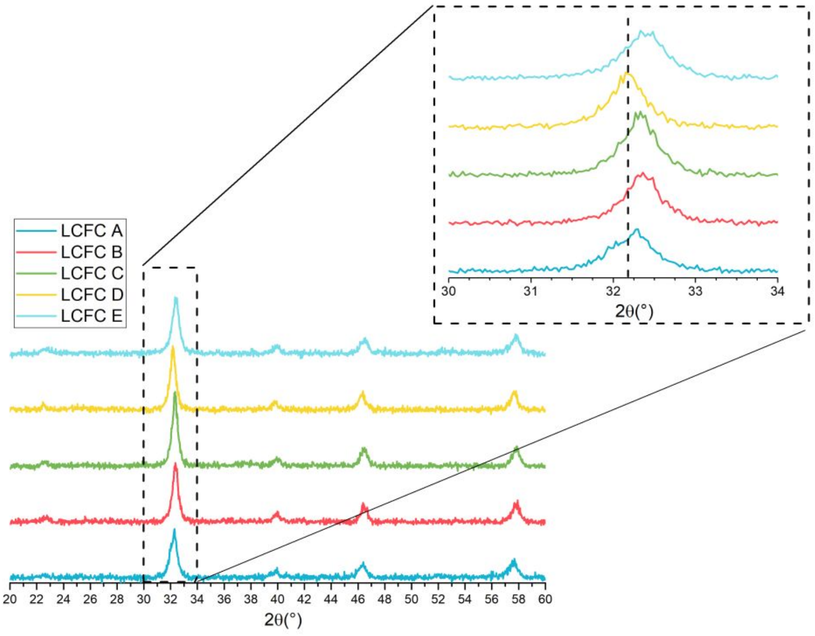

Figure S5. XRD pattern for LCFC A, B, C, D and E. Inset: the most intense peak.

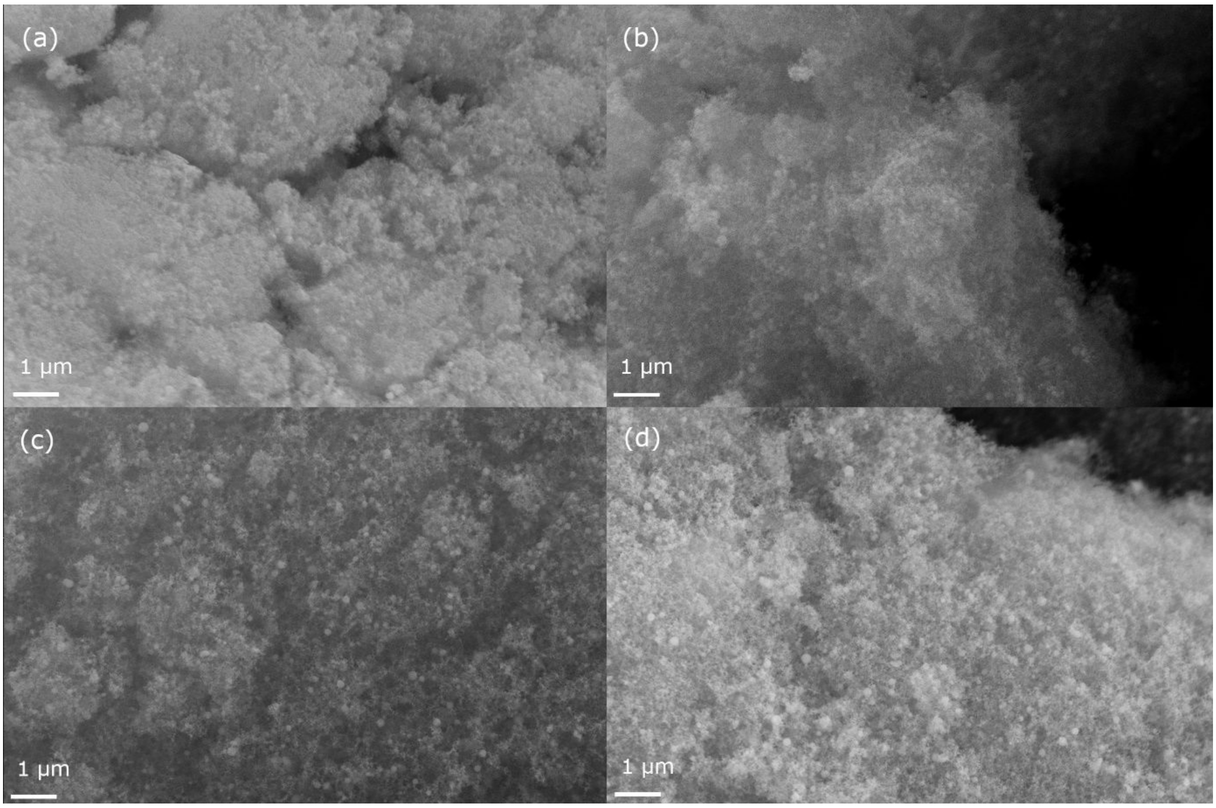

Figure S6. SEM images for LCFC A sample (a), LCFC B (b), LCFC CC (c), LCFC D (d).

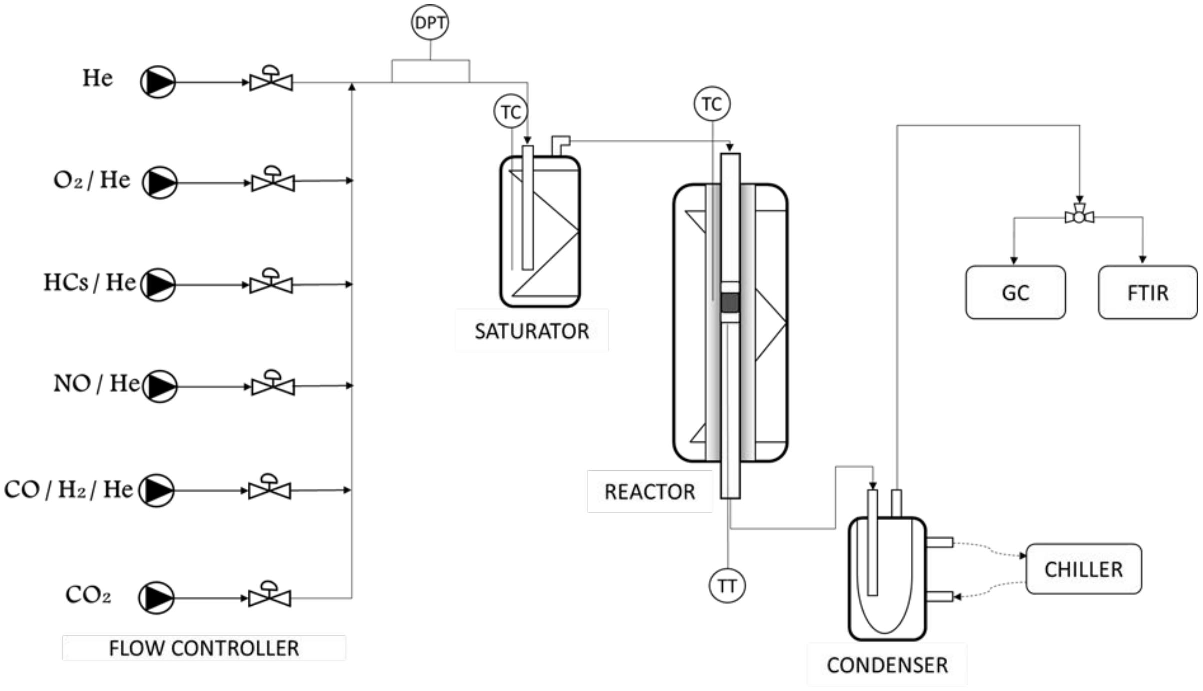

Figure S7. Catalytic setup for complex mixture testing.

Sample	*Type*	*La*	*Ca*	*Fe*	*Cu*	*O*	*La**	*Ca**	*Fe**	*Cu**	*Ca/La*	*Cu/Fe*	*(Fe+Cu)/ (La+Ca)*	*O/ (La+Ca+Fe+Cu)*	*O lattice/O surface*[a]
LCFC A	XPS	14.4	2.6	15.7	2.2	65.0	41.2	7.5	44.9	6.4	0.18	0.14	1.05	1.86	5.5
LCFC B	XPS	14.3	2.5	18.5	3.5	61.1	36.8	6.5	47.4	8.9	0.18	0.19	1.30	1.57	1.0
LCFC C	XPS	15.0	3.8	17.2	3.6	60.4	37.9	9.6	43.5	9.0	0.25	0.21	1.11	1.53	2.1
LCFC D	XPS	13.9	3.0	19.2	3.7	60.3	35.0	7.5	48.4	9.2	0.21	0.19	1.36	1.52	1.3
LCFC E	XPS	15.8	2.3	17.0	0.9	63.9	43.9	6.4	47.2	2.5	0.15	0.05	0.99	1.77	1.9
LCFC COP	XPS	16.3	2.2	20.0	3.2	58.3	39.0	5.3	48.0	7.6	0.14	0.16	1.25	1.40	3.6
LFC FSP	XPS	13.9		17.1	3.6	65.4	40.1		49.5	10.4		0.21	1.49	1.89	0.2
LCFC	Nominal	12.5	4.2	16.6	4.2	62.5	33.0	11.0	44.0	11.0	0.33	0.3	1.22	1.67	
LFC	Nominal	14.9		17.0	4.3	63.8	35.0		40.0	25.0		0.6	1.86	1.76	

Sample	*Type*	*La*	*Ca*	*Fe*	*Cu*	*O*	*La**	*Ca**	*Fe**	*Cu**	*Ca/La*	*Cu/Fe*	*(Fe+Cu)/ (La+Ca)*	*O/ (La+Ca+Fe+Cu)*	*O lattice/O surface*[a]
LCFC A	EDX	8.0	2.2	9.9	1.2	78.8	37.8	10.2	46.6	5.8	0.27	0.12	1.09	3.72	
LCFC B	EDX	8.3	2.7	11.3	1.7	76.1	34.7	11.2	47.1	6.9	0.32	0.15	1.18	3.18	
LCFC C	EDX	11.0	3.2	14.4	1.9	69.6	36.1	10.4	47.4	6.1	0.29	0.13	1.15	2.29	
LCFC D	EDX	9.8	2.9	12.6	1.5	73.3	36.5	11.0	47.1	5.4	0.30	0.12	1.11	2.75	
LCFC E	EDX	10.7	2.9	12.9	1.6	71.9	38.0	10.4	45.9	5.6	0.27	0.12	1.06	2.56	
LCFC COP	EDX	12.9	1.4	12.9	3.6	69.2	41.9	4.5	41.9	11.7	0.11	0.28	1.16	2.25	
LFC FSP	EDX	12.1		14.6	1.8	71.5	42.4		51.2	6.4		0.13	1.36	2.51	
LCFC	Nominal	12.5	4.2	16.6	4.2	62.5	33.0	11.0	44.0	11.0	0.33	0.3	1.22	1.67	
LFC	Nominal	14.9		17.0	4.3	63.8	35.0		40.0	25.0		0.6	1.86	1.76	

Table S2. Compositions. XPS and EDX compositions (atomic concentrations) obtained for the doped and un-doped LFC obtained by Flame Spray Pyrolysis (FSP) and by Co-precipitation (COP). The compositions obtained without considering oxygen (*) are reported in order to emphasize the cation surface segregation phenomena. The last column refers to the ratio between the integrated peak area contribution at 529 eV and the integrated peak area contribution at 532 eV.

ICP analytic results

Generally the experimental composition approaches more the nominal one for the samples obtained by COP method. In the first Table (Table S3) the samples collected at different phases of the FSP process are progressively converging to the nominal throughout the process, however La never reaches the nominal composition.

In the second Table LFC FSP shows that the copper content is roughly half of the nominal expected, meaning that copper is not perfectly included in the structure. Unlike Cu, La and Fe are in agreement with the expected results. Comparing LCFC obtained by COP with the analogous samples by FSP, COP sample includes Cu better than FSP, whereas Ca has been included in a lower amount that expected.

		ICP results (%)					
	Nominal	LCFC A	LCFC B	LCFC C	LCFC D	LCFC E	LCFC COP
La	54.26	61.95	58.63	59.99	62.07	61.75	60.62
Ca	8.34	4.08	4.75	5.99	4.48	4.22	2.17
Cu	8.29	3.99	5.88	3.77	3.66	4.06	7.99
Fe	29.12	29.98	30.74	30.25	29.78	29.97	29.22

		ICP results (%)
	Nominal	LFC FSP
La	62.93	64.25
Cu	8.22	4.59
Fe	28.85	31.16

Table S3. ICP composition of the samples compared to the nominal composition (only metal).

Catalytic activity

	Temperature of half conversion (°C) commercial sample	Temperature of half conversion (°C) LCFC A	Temperature of half conversion (°C) LCFC C	Temperature of half conversion (°C) LCFC E	Temperature of half conversion (°C) LCFC COP	Temperature of half conversion (°C) LFC FSP
CO conversion	217	235	305	240	242	211
C_3H_6 conversion	262	378	410	398	366	331
NO conversion	455	326	352	329	289	327

Table S4. Comparison with a commercial catalyst (Data taken from [8]), stoichiometric complex mixture.

Each of the five FSP samples obtained under different production conditions, have been tested in CO assisted NO reduction (Figure 8). Samples A, C, and E have also been tested with complex mixtures (Figures S8 and S9) for stoichiometric and rich mixture, respectively), given their remarkable differences in properties as revealed by the structural and compositional analysis.

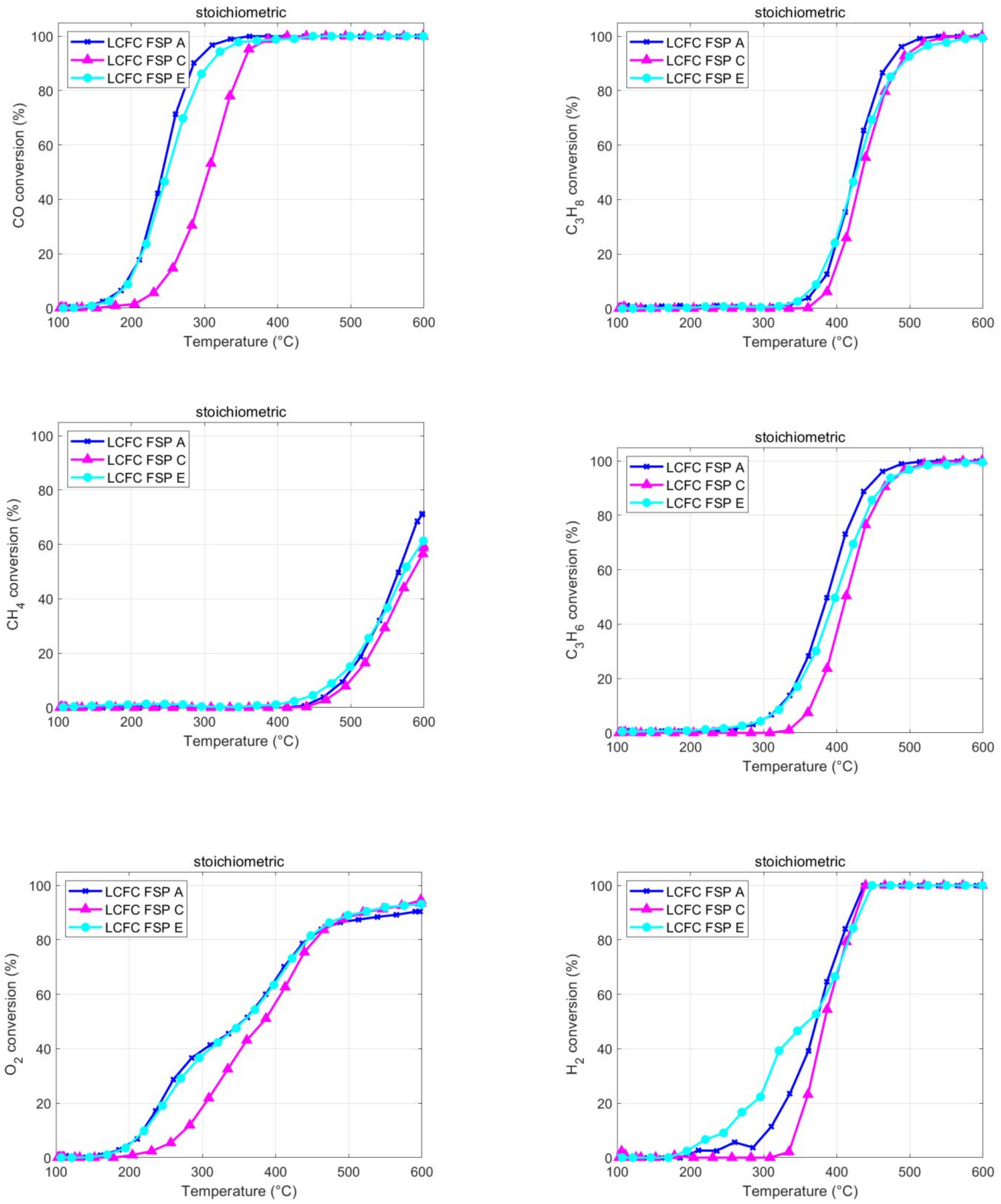

Figure S8. Activity of LCFC FSP A, C, and E. Stoichiometric TWC mixture.

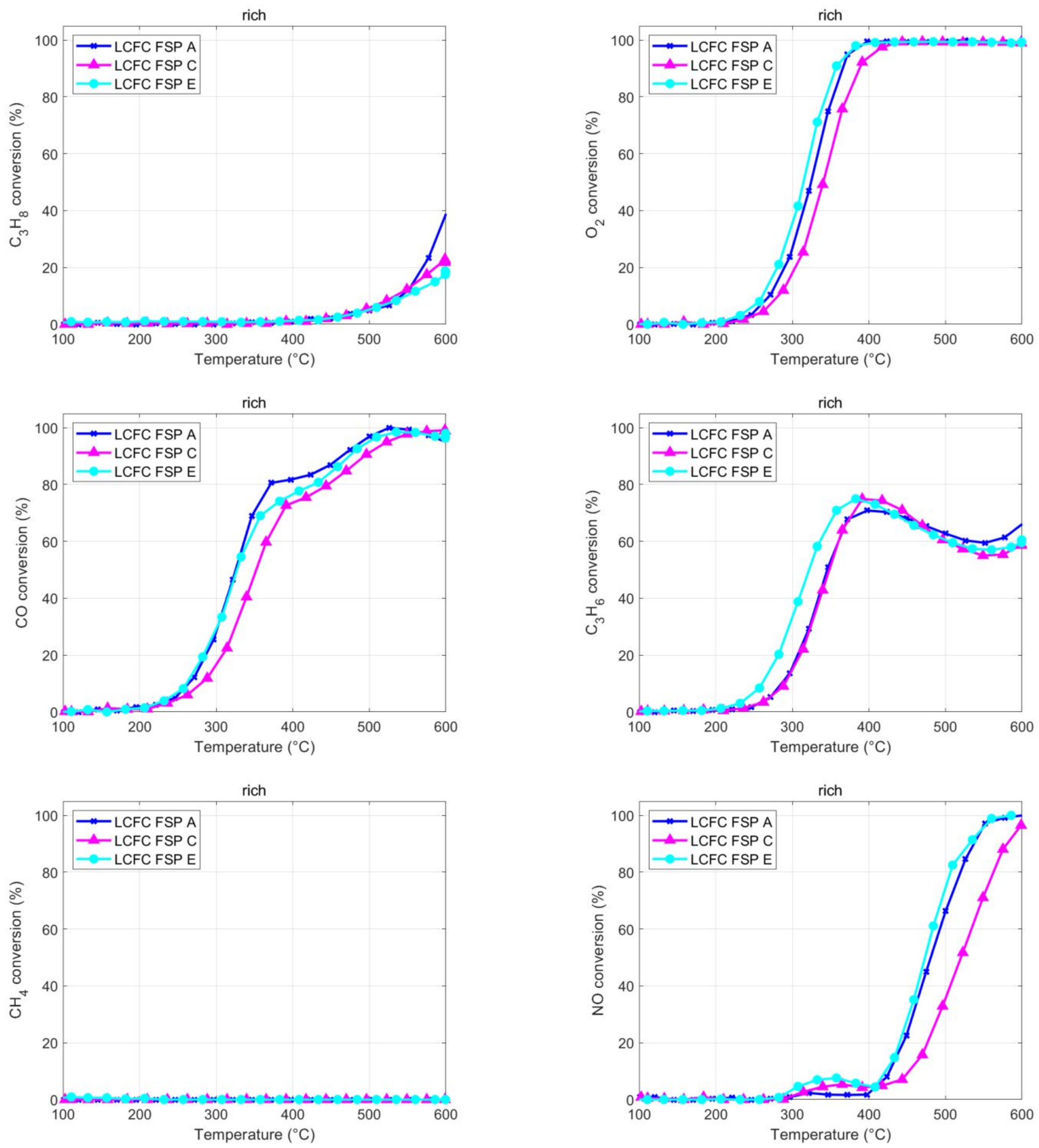

Figure S9. Activity of LFC FSP A, C, and E. Rich TWC mixture.

(1) Garbujo, A.; Pacella, M.; Natile, M. M.; Guiotto, M.; Fabro, J.; Canu, P.; Glisenti, A. On A-Doping Strategy for Tuning the TWC Catalytic Performance of Perovskite Based Catalysts. *Appl. Catal. A Gen.* **2017**, *544* (July), 94–107. https://doi.org/10.1016/j.apcata.2017.07.009.

(2) Naumkin, V. A.; Kraut-Vass, A.; Gaarenstroom, S. W.; J., P. C. NIST X-Ray Photoelectron Spectroscopy Database. *Meas. Serv. Div. Natl. Inst. Stand. Technol.* **2012**, *20899* (20), 20899. https://doi.org/10.18434/T4T88K.

(3) McIntyre, N. S.; Zetaruk, D. G. X-Ray Photoelectron Spectroscopic Studies of Iron Oxides. *Anal. Chem.* **1977**, *49* (11), 1521–1529. https://doi.org/10.1021/ac50019a016.

(4) Glisenti, A.; Pacella, M.; Guiotto, M.; Natile, M. M.; Canu, P. Largely Cu-Doped LaCo1-XCuxO3 Perovskites for TWC: Toward New PGM-Free Catalysts. *Appl. Catal. B Environ.* **2016**, *180*, 94–105. https://doi.org/10.1016/j.apcatb.2015.06.017.

(5) Sorenson, S. C.; Wronkiewicz, J. A.; Sis, L. B.; Wirtz, G. P. Properties of LaCoO3 as a Catalyst in Engine Exhaust Gases. *Ceram. Bull.* **1974**, *53* (5), 446–449.

(6) Rossetti, I.; Biffi, C.; Forni, L. Oxygen Non-Stoichiometry in Perovskitic Catalysts: Impact on Activity for the Flameless Combustion of Methane. *Chem. Eng. J.* **2010**, *162* (2), 768–775. https://doi.org/10.1016/j.cej.2010.06.003.

(7) Isupova, L. A.; Yakovleva, I. S.; Rogov, V. A.; Alikina, G. M.; Sadykov, V. A. Oxygen States in Oxides with a Perovskite Structure and Their Catalytic Activity in Complete Oxidation Reactions: System La 1 - XCa XFeO 3 - y (x = 0-1). *Kinet. Catal.* **2004**, *45* (3), 446–453. https://doi.org/10.1023/B:KICA.0000032182.48410.e2.

(8) Schön, A.; Dacquin, J. P.; Granger, P.; Dujardin, C. Non Stoichiometric La1-YFeO3 Perovskite-Based Catalysts as Alternative to Commercial Three-Way-Catalysts? – Impact of Cu and Rh Doping. *Appl. Catal. B Environ.* **2018**, *223*, 167–176. https://doi.org/10.1016/j.apcatb.2017.06.026.

(9) Umbach, E. *Practical Surface Analysis*; Wiley: New York, 1992; Vol. 11. https://doi.org/10.1016/0165-9936(92)87016-d.
